# Varicella Viruses Inhibit Interferon-Stimulated JAK-STAT Signaling through Multiple Mechanisms

**DOI:** 10.1371/journal.ppat.1004901

**Published:** 2015-05-14

**Authors:** Marieke C. Verweij, Mary Wellish, Travis Whitmer, Daniel Malouli, Martin Lapel, Stipan Jonjić, Juergen G. Haas, Victor R. DeFilippis, Ravi Mahalingam, Klaus Früh

**Affiliations:** 1 Vaccine and Gene Therapy Institute, Oregon Health and Science University, Beaverton, Oregon, United States of America; 2 Department of Neurology, University of Colorado School of Medicine, Aurora, Colorado, United States of America; 3 Department of Histology and Embryology, Faculty of Medicine, University of Rijeka, Rijeka, Croatia; 4 Division of Infection and Pathway Medicine, University of Edinburgh, Edinburgh, United Kingdom; University of Glasgow, UNITED KINGDOM

## Abstract

Varicella zoster virus (VZV) causes chickenpox in humans and, subsequently, establishes latency in the sensory ganglia from where it reactivates to cause herpes zoster. Infection of rhesus macaques with simian varicella virus (SVV) recapitulates VZV pathogenesis in humans thus representing a suitable animal model for VZV infection. While the type I interferon (IFN) response has been shown to affect VZV replication, the virus employs counter mechanisms to prevent the induction of anti-viral IFN stimulated genes (ISG). Here, we demonstrate that SVV inhibits type I IFN-activated signal transduction via the JAK-STAT pathway. SVV-infected rhesus fibroblasts were refractory to IFN stimulation displaying reduced protein levels of IRF9 and lacking STAT2 phosphorylation. Since previous work implicated involvement of the VZV immediate early gene product ORF63 in preventing ISG-induction we studied the role of SVV ORF63 in generating resistance to IFN treatment. Interestingly, SVV ORF63 did not affect STAT2 phosphorylation but caused IRF9 degradation in a proteasome-dependent manner, suggesting that SVV employs multiple mechanisms to counteract the effect of IFN. Control of SVV ORF63 protein levels via fusion to a dihydrofolate reductase (DHFR)-degradation domain additionally confirmed its requirement for viral replication. Our results also show a prominent reduction of IRF9 and inhibition of STAT2 phosphorylation in VZV-infected cells. In addition, cells expressing VZV ORF63 blocked IFN-stimulation and displayed reduced levels of the IRF9 protein. Taken together, our data suggest that varicella ORF63 prevents ISG-induction both directly via IRF9 degradation and indirectly via transcriptional control of viral proteins that interfere with STAT2 phosphorylation. SVV and VZV thus encode multiple viral gene products that tightly control IFN-induced anti-viral responses.

## Author Summary

In this manuscript we demonstrate that the immediate early protein ORF63 encoded by varicella zoster virus (VZV) and simian varicella virus (SVV) interferes with interferon type I-mediated activation of JAK-STAT signaling and thereby inhibits the expression of interferon stimulated genes. ORF63 blocks this pathway by degrading IRF9, which plays a central role in JAK-STAT signaling. In addition, both viruses code for immune evasion mechanisms affecting the JAK-STAT pathway upstream of IRF9, which results in the inhibition of STAT2 phosphorylation. By fusing a degradation domain derived from dihydrofolate reductase (DHFR) to ORF63 we further demonstrate that this protein is essential for SVV growth and gene expression, indicating that ORF63 also affects IFN-signaling indirectly by regulating the expression of other immune evasion genes.

## Introduction

The alphaherpesvirus varicella zoster virus (VZV) is the causative agent of chickenpox. After primary infection, VZV establishes latency in sensory ganglia. Reactivation from latency, which typically occurs in elderly individuals, can cause shingles or herpes zoster that is associated with a number of debilitating complications, including postherpetic neuralgia [1]. *In vivo* research on VZV is limited because the virus does not produce varicella or zoster in animals [2, 3]. Simian varicella virus (SVV) is closely related to VZV sharing about 75% DNA homology and exhibiting a highly similar genome organization [4]. Inoculation of nonhuman primates, including African green monkeys and Cynomolgus macaques, results in a persistent viremia [4]. In contrast, infection of rhesus macaques (RM) with SVV results in a primary infection followed by latency that is similar to VZV infection in humans. SVV-induced skin lesions are resolved by 21 days post infection which correlates with the absence of virus DNA in blood. Latent SVV can be detected in ganglia of infected RM [5]. Infection of RM with SVV thus represents a robust animal model that recapitulates most hallmarks of a primary human VZV infection.

The innate host immune response to viral infection is dominated by interferons (IFNs) that are subdivided in three families, namely types I, II and III. In particular, several subtypes of IFNα and IFNβ that represent type I IFNs are key players in the anti-viral innate immune response [6]. Transcription of IFN is initiated by pattern recognition receptors (PRRs) engaging pathogen associated molecular patterns (PAMPs) such as double-stranded RNA, lipopolysaccharide and cytosolic DNA. Downstream signaling pathways lead to the activation of transcription factors such as IFN regulatory factor (IRF) 3 and nuclear factor κB (NFκB) that induce the transcription of IFNβ. Secreted IFNβ can signal in an autocrine and paracrine fashion by interacting with the type I IFN receptor complex (consisting of IFNAR1 and IFNAR2) both on infected and neighboring uninfected cells [7]. Receptor binding activates the JAK-STAT signaling pathway, which results in the expression of hundreds of IFN-stimulated genes (ISG) and corresponding proteins that inhibit virus growth by counteracting multiple molecular steps of the replication cycle and by signaling to innate immune cells including natural killer cells [8, 9]. In addition, type I IFNs have been shown to be involved in dendritic cell maturation and antigen presentation thereby stimulating the development of virus-specific adaptive immune responses [10, 11].

The ability of VZV and SVV to spread and establish latency in the presence of these immediate immune responses implies that both viruses display evasion strategies that circumvent or counteract the induction or function of IFNs and ISGs. VZV infection of human skin xenografts in severe combined immunodeficiency (SCIDhu) mice showed that VZV-infected cells do not express type I IFN, while uninfected bystander cells stained positive for the cytokine [12]. Several reports have shown that the effect of IFN is counteracted by at least four different VZV-encoded proteins: both IE62 and ORF47 alter the phosphorylation of IRF3 and prevent gene activation [13, 14], whereas ORF61 inhibits pathogen-induced cytokine expression by degrading IRF3 [15] and by blocking the activation of NFκB via inhibiting IκBα [16]. In addition, Cohen *et al*. showed the deletion of ORF63 severely attenuates the growth of the virus in the presence of IFNα but not IFNγ, suggesting a possible involvement of ORF63 in regulating JAK-STAT signaling [17].

Expression of IFNα and IFNβ and subsequent binding to their receptor triggers the dimerization of IFNAR1 and IFNAR2, which activates the Janus kinases JAK1 and TYK2 that are constitutively associated with the receptor. The kinases phosphorylate the receptor creating a docking site for the transcription factors STAT1 and STAT2. Subsequent phosphorylation of the STAT proteins by the JAKs leads to conformational changes within the molecules that allow the formation of stable STAT1, STAT2 and IFN regulatory factor 9 (IRF9) complex termed ISGF3. ISGF3 then shuttles to the nucleus where it activates the transcription of type I IFNs and ISGs [18]. VZV-infected cells in skin xenografts in the SCIDhu mice did not show nuclear localization or phosphorylation of STAT1, in contrast to uninfected bystander cells [12]. Since IFNα produced by bystander cells can induce JAK-STAT signaling in VZV-infected cells, the absence of pSTAT1 in infected cells suggests that VZV interferes not only with IRF3-mediated activation of IFN transcription but also with JAK/STAT signaling.

The VZV ORF63 protein is a 30 kDa immediate early protein that is phosphorylated by host and viral kinases [19, 20]. The protein is abundantly present during lytic infection and its expression has also been observed in latently infected ganglia [21, 22]. A duplicate of the ORF63 gene, designated ORF70, is found in the terminal repeat region [19]. VZV ORF63/70 regulates viral gene expression and impairs the expression of certain cellular genes [23–26]. In addition, VZV ORF63/70 inhibits apoptosis in cultured primary human neurons [27]. SVV encodes both ORF63/70 orthologous proteins that share 52% amino acid identity with their VZV homologs [28]. These gene products are required for replication of SVV in cell culture [29]. *In vivo* studies using SVV-infected RM and African green monkeys confirmed expression of the ORF63 protein in ganglia during latent infection [5].

In this report, we show that both SVV and VZV interfere with type I IFN signaling. SVV inhibits type I IFN-induced ISG expression by downregulating the expression of IRF9 and prevents phosphorylation of STAT2 upon IFN stimulation. We also observed a minor decrease in STAT2 levels in SVV-infected cells. SVV ORF63 was found to be responsible for the reduction in IRF9 expression, but was not directly involved in downregulation of STAT2 expression or inhibition of STAT2 phosphorylation. These data suggest that multiple SVV proteins counteract type I IFN signaling. Similarly, we observed reduced levels of STAT2 and IRF9 proteins in VZV-infected cells and the cells were refractory to IFN-induced STAT2 phosphorylation. Ectopic expression of VZV ORF63 affected steady state levels of IRF9, but did not block STAT2 expression or phosphorylation. Thus, the multi-level inhibition of JAK-STAT signaling seen in SVV-infected cells is conserved in VZV-infected cells.

## Materials and Methods

### Cell lines and recombinant viruses

Telomerized rhesus fibroblasts (TRFs), TRF-ISRE cells [30], the African green monkey kidney epithelial cell line Vero, Vero-CRE (kindly provided by Dr. Linda van Dyk, University of Colorado, Denver) [31], telomerized human fibroblasts (THF)-ISRE [32], human embryonic kidney (HEK) 293T cells (ATCC), and the human fibroblast cell line MRC-5 (ATCC) were maintained in DMEM supplemented with 10% heat-inactivated fetal bovine serum (FBS), 140 IU of penicillin/ml and 140 μg of streptomycin/ml.

TRFs and Vero cells were infected with a recombinant SVV Delta strain, in which eGFP was inserted between US2 and US3 through homologous recombination [33]. A confluent monolayer of TRF or Vero cells was infected by cocultivation of SVV.eGFP-infected cells with uninfected cells at the indicated ratios. Complete infection was confirmed by visualizing eGFP using fluorescence microscopy. Infected cells were maintained in DMEM supplemented with 2% FBS and harvested at 48 hours p.i. For VZV infections, we used the recombinant VZV Oka strain, in which eGFP was fused to the N-terminus of ORF66 (generously provided by P.R. Kinchington, University of Pittsburgh, Pennsylvania) [34]. VZV.eGFP-infected MRC-5 cells were cocultivated with uninfected MRC-5 cells at a 1:5 ratio.

### Reagents and antibodies

Rhesus IFNα2 was obtained from R&D systems. Human IFNα and universal type I IFN (uIFN) were obtained from PBL Assay Science. MG132 (Fisher Scientific) was dissolved in DMSO and used at the indicated concentrations for 16 hours. Control wells were treated with same concentration of DMSO without MG132. Trimethoprim (TMP; Sigma-Aldrich) was dissolved in DMSO and used 10 μM or less where indicated. Viral cultures were supplemented with fresh TMP every 24 hours. The following antibodies were used for detection of endogenous and viral proteins in western blot: anti-ISG15 F-9 (Santa Cruz), anti-ISG54/IFIT2 (Abcam), anti-Mx-1 (GeneTex), anti-STAT1 M22 (Santa Cruz), anti-phosphorylated STAT1 Tyr701 (Santa Cruz), anti-STAT2 C20 (Santa Cruz), anti-phosphorylated STAT2 Tyr690 (Cell Signaling Technology), anti-IRF9/ISGF3γ clone 6 (BD Biosciences), anti-GAPDH 6C5 (Santa Cruz), anti-p84 5E10 (GeneTex), anti-IRF1 H-205 (Santa Cruz), anti-IRF3 (Santa Cruz) and anti-FLAG M2 (Sigma-Aldrich). The monoclonal antibodies specific for SVV and VZV ORF63 (clone 63_6), ORF62 (clone 62_6) and ORF31 (clone 31C_8) have been previously described [35]. STAT2 C20 (Santa Cruz) was also used for immunofluorescence microscopy.

### Luciferase reporter assays

**Fig 1:**
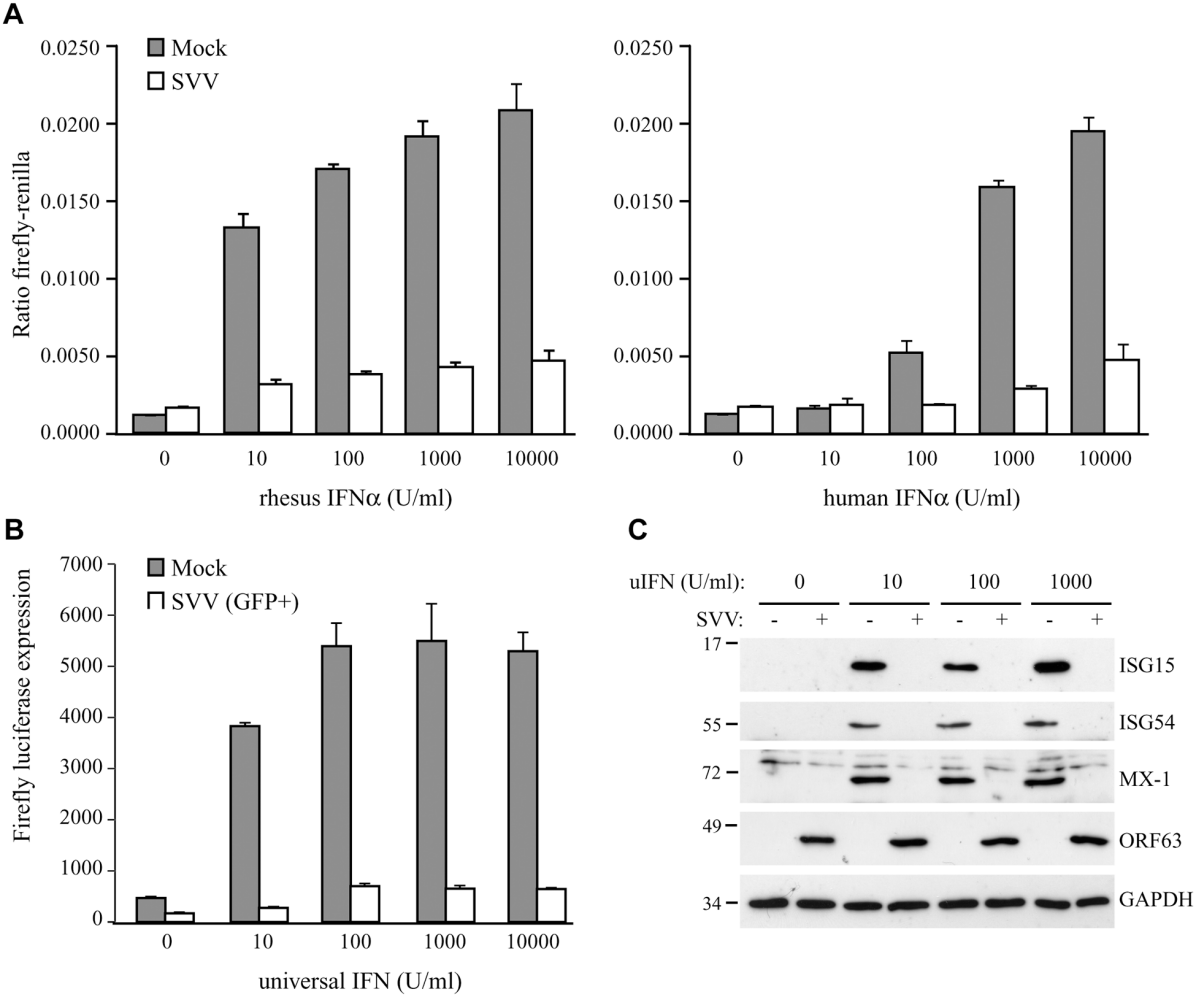
SVV inhibits IFN-induced ISG expression. TRFs stably expressing firefly luciferase under an ISRE promotor and constitutively expressing renilla luciferase (TRF-ISRE) were mock infected or infected with SVV.eGFP in a ratio of 5:1. (A) At 42 hours p.i. the cells were stimulated with increasing concentrations of rhesus (upper panel) or human (lower panel) IFNα for 6 hours, after which luciferase expression was measured. ISRE promotor activity was normalized to renilla luciferase. (B) At 40 hours p.i. cells expressing high GFP levels were sorted by flow cytometry and subsequently stimulated with increasing concentrations of uIFNα for 6 hours, after which firefly luciferase expression was measured. (C) TRFs were infected with SVV.eGFP in a ratio of 5:1 and at 40 hours p.i. the cells were stimulated with the indicated concentration of uIFN for 8 hours. Cell lysates were analyzed for ISG protein expression by SDS-PAGE and western blotting using specific antibodies. Productive SVV infection was confirmed using an antibody specific for SVV ORF63 and the GAPDH signal was used a loading control. Results for one of three independent experiments is shown.

TRFs were transduced with a replication-defective lentivirus encoding firefly luciferase downstream of an IFN-stimulated response element (ISRE) and a lentivirus constitutively expressing renilla luciferase driven by a CMV promotor (Qiagen). Transduced TRFs (TRF-ISRE cells) were selected by culturing in the presence of 4 μg/ml puromycin. TRF-ISRE cells were infected with SVV.eGFP as described above and 24 hours p.i. the cells were seeded in a black 96 well plate (Corning Incorporated). At 42 hours p.i., the cells were stimulated with rhesus or human IFNα to induce expression of ISRE-driven firefly luciferase. After 6 hours, expression of firefly and renilla luciferase was measured using the Dual-Glo luciferase assay system (Promega). Luminescence was measured on a Veritas microplate luminometer (Promega). Data are presented as the ratio between firefly luciferase expression and renilla luciferase expression. For the experiment described in Fig 1B the cells were sorted for high GFP expression at 40 hours p.i. using a FACS Aria II cytometer. We seeded 2000 of the sorted and mock-infected cells cells in black 96 well plate and stimulated with uIFN for 6 hours, after which luciferase expression was measured using the ONE-Glo luciferase assay system (Promega).

HEK 293T cells were co-transfected with an ISRE-firefly luciferase reporter plasmid (pGL3-ISRE-Luc) and the pcDNA3.1 expression vectors (described below). At 24 and 42 hours post transfection, the cells were treated with 5000 U/ml uIFN for 6 hours. Cells were transferred to a black 96 wells plate before or right after treatment with uIFN treatment. Expression of firefly luciferase was measured using the ONE-Glo luciferase assay system (Promega).

### Nuclear extractions and Western blotting

To generate nuclear and cytoplasmic fractions, cells were resuspended in dounce buffer (100 mM KCl, 20 mM Hepes [pH 7.4], 0.1 mM EDTA, 3% sucrose) supplemented with HALT protease and phosphatase inhibitors (Thermo Scientific). After 15 minutes 10% Nonidet P-40 was added to the lysate in a 1:20 ratio and lysates were vortexed for 10 seconds. The cytoplasmic fraction was removed immediately and nuclei were washed twice with D-PBS and lysed in RIPA buffer (10 mM Tris-HCl [pH 7.4], 150 mM NaCl, 1 mM EDTA, 1% Triton X-100, 1% Sodium Deoxycholate, 0.1% SDS) supplemented with protease and phosphatase inhibitors. For all other experiments, cells were lysed directly in Laemmli sample buffer (100 mM Tris-HCL [pH 8.0], 4% SDS, 20% glycerol, 10% 2-mercaptoethanol, Bromophenol blue). Proteins were separated by SDS-page and transferred to polyvinylidene difluoride membranes (Thermo Scientific). Membranes were first incubated with the indicated antibodies, which was followed by incubation with horseradish peroxidase (HRP)-conjugated secondary antibodies specific for mouse (Santa Cruz) or rabbit (Thermo Scientific) IgG. Binding of secondary antibodies to the membranes was visualized by using Pierce ECL 2 (Thermo Scientific).

### Immunofluorescence microscopy

Mock- or SVV-infected TRFs were grown on cover slips, washed twice with PBS and fixed with 3.7% formaldehyde (Fisher Scientific) at room temperature (RT) for 40 minutes. After washing with PBS, residual formaldehyde was quenched with 50 mM Ammonium Chloride for 10 minutes and the cells were permeabilized with 0.1% Triton for 5 to 7 minutes. Non-specific protein binding sites were blocked with 2% bovine serum albumin (BSA) (Fisher Scientific) and cells were incubated with STAT2-specific antibody in 2% BSA for 1 hour at 37°C. Cells were washed with 2% BSA and incubated with the secondary antibody Alexa Fluor 594 Goat anti-Rabbit (Life Technologies). Cells were then washed with 2% BSA, followed by one PBS wash. Cover slips were mounted on glass slides using Prolong Gold Anti-fade reagent (Cell Signaling). Staining was visualized on a Zeiss Axioskop 2 Plus fluorescence microscope, and images were taken using AxioVision v4.6 software (Zeiss).

### Adenovirus production and infection

The recombinant adenoviruses expressing SVV ORF63 (AdORF63) and VZV AdORF63 were produced as previously described [36]. The vector contains a tetracycline-responsive promoter and requires the addition of a tetracycline-regulated transactivator (tTA) [37], which was provided by co-infecting with AdtTA. TRFs cultured in six-well clusters were co-transduced with the purified ORF63 adenoviruses and AdtTA at the indicated MOI in 0.5 ml of serum-free DMEM. After 1.5 hour of incubation at 37°C, 1.5 ml of DMEM supplemented with 10% FBS was added and incubation continued for a total of 48 hours. Where indicated, doxycycline was added to the infections to regulate tTA-dependent gene expression.

### RNA isolation and semiquantative PCR

Total RNA was extractedand treated with DNase using the NucleoSpin RNA isolation kit (Machery Nagel) according to the manufacturer's protocol. The concentration of the RNA samples was measured using the NanoDrop 1000 Spectrophotometer (Thermo Scientific). Single-stranded cDNA was made from total RNA using random hexamers (TaKaRa) to prime first-strand synthesis by Maxima Reverse Transcriptase (Thermo Scientific) as recommended by the manufacturer. The induction of ISG54 and Mx-1 mRNA expression upon uIFN stimulation was determined using SYBR green-based semiquantitative real-time RT-PCR (qPCR) using the following primers: ISG54 Fw: 5’-gttactggaactaataggacac-3’, ISG54 Rev: 5’-tggcaagaatggaaca-3’, Mx-1 Fw: 5’-atgatcgtcaagtgccg-3’, Mx-1 Rev: 5’-gccttgccttcctcca-3’. SVV ORF63 expression was confirmed using the primers ORF63 Fw: 5’-CAGCGTCCTACAGTGAC-3’ and ORF63 Rev: 5’-GTTGCTGGTAGCATCATC-3’. Levels of IRF9 mRNA were determined using the primers IRF9 Fw: 5’- TACCCGAAAACTCCGGAAC-3’ and IRF9 Rev: 5’-AAGAAGGCAGCATCCTGG-3’. Levels of STAT2 mRNA were determined using the primers STAT2 Fw: 5’- ATGGCGCAGTGGGAAATG-3’ and STAT2 Rev: 5’-ctgccagttctggtcttc-3’. Reactions were performed using SYBR green PCR core reagents and Platinum Taq DNA Polymerase (Invitrogen). Relative expression of ISG54, Mx-1, and ORF63 was calculated using the method described by Livak and Schmittgen [38]. GAPDH was used as a housekeeping gene to establish a baseline against which target genes were compared (Fw: 5’-GCACCACCAACTGCTTAGCAC-3’, Rev: 5’- TCTTCTGGGTGGCAGTGATG-3’). For IRF9 and STAT2 mRNA expression we calculated delta cycle threshold (ΔCt) by subtracting background Ct (GAPDH) from the Ct value for IRF9/STAT2.

### Plasmids and transient transfections

Purified DNA from TRFs infected with SVV.eGFP was used as a template for PCR amplification of SVV ORF63. PCR was performed using AccuPrime Taq DNA polymerase High Fidelity (Life Technologies) using the primers 5’-AATAAAGGATCCGCCACCATGCAGGCGCCCCGAG-3’ (Fw) and 5’-AATAAAGAATTCTTATGTATTGTGTACAGACTCTCGTAACTCCGTG-3’ (Rev) to amplify the coding sequence of the SVV ORF63 gene. The PCR-generated product was inserted into pcDNA3.1-IRES-nlsGFP using BamHI/EcoRI sites, creating pcDNA3.1 ORF63. To create pcDNA3.1 FLAG-ORF32 we amplified ORF32 from the same template with the primers 5'-(AATAAAGGATCCGCCACCATGGCATCATCTAATACTTGCGAAGAACAAAATAATTCTA)-3' (Fw) and 5'-(AATAAAGAATTCTTActtatcgtcgtcatccttgtagtcATCCGTTTCGCTCTCGCTAGATGAAGGTTG)-3' (Rev) using the Expand High Fidelity PCR system (Roche). The PCR-generated product was inserted into the pcDNA3.1-IRES-nlsGFP vector using BamHI/EcoRI sites. VZV ORF63 was amplified from DNA extracted from VZV.eGFP-infected MRC-5 cells using the Expand Expand High Fidelity PCR system (Roche) and the following primers: 5'-(AATAAAGGATCCGCCACCATGTTTTGCACCTCACCGGC)-3' (Fw) and 5'-(AATAAAGAATTCCTACACGCCATGGGGGGGCGGTATATC)-3' (Rev). The resulting insert was cloned into pcDNA3.1-IRES-nlsGFP using BamHI/EcoRI sites. Rhesus IRF9 was synthesized and codon-optimized for expression in rhesus cell lines by GenScript. The insert was cloned from the pUC57 plasmid into pcDNA3.1-IRES-nlsGFP using BamHI/EcoRI restriction sites, creating pcDNA3.1 IRF9. All ligations were performed using the Rapid DNA Dephos and Ligation kit (Roche). The DNA sequences of all expression plasmids were verified.

HEK 293T cells were transfected with the indicated plasmids and pGL3-ISRE-Luc (a kind gift from Dr. John Hiscott, Vaccine and Gene Therapy Institute, Florida) using the Lipofectamine 2000 reagent (Life Technologies) using the manufacturers protocol. For the IRF9 overexpression experiment we used 1 μg pGL3-ISRE-Luc and the indicated amounts pcDNA3.1 (p)ORF63 and pcDNA3.1 (p)IRF9. Control pcDNA3.1 or pRetro-E2 expressing GFP was used to equalize all transfection samples to a total of 6 μg DNA. Transfection efficiency was confirmed measuring GFP expression in all samples using Synergy HTX Multi-Mode Reader (Bio-Tek) or by staining for specific proteins in western blot. To test whether VZV ORF63 inhibits IFN-signaling, we used 1 μg of pGL3-ISRE-Luc and 3 μg of the other indicated plasmids. 48 hours post transfection IFN-signaling was assessed using a luciferase assay, described below.

### Lentivirus construction and infection

GIPZ lentivirus constructs expressing shRNA specific for human IRF9 were obtained from Open Biosystems/GE Healthcare. The constructs used are V3LHS-322329 (shRNA-1), V3LHS-322332 (shRNA-2), and V2LHS-69847 (shRNA-3). Replication deficient lentiviruses were produced by transfecting the shRNA vectors into HEK 293T cells and providing the vesicular stomatitis virus G (pMD2.G VSV-G, Addgene) protein and the packaging plasmid psPAX2 (Addgene) in trans. The plasmids were transfected using Lipofectamine LTX (Life Technologies). 48 hours post transfection the supernatant containing lentivirus was harvested and transferred to target cells, which were transduced in the presence of 5 μg/ml Polybrene (Hexadimethrine bromide; Sigma-Aldrich). After 24 hours, this process was repeated. The resulting cell lines were grown in the presence of 3 μg/ml Puromycin to select for shRNA expressing cells.

### Statistical analysis

P-values were determined using unpaired Student’s t-test.

### SVV BAC mutagenesis

To prepare the SVV ORF63/70 mutant, we used an SVV BAC containing the complete SVV genome and eGFP driven by the CMV immediate-early promoter [39]. To introduce mutations into SVV ORF63/70, we used the two-step red-mediated mutagenesis protocol [40]. Mutagenesis of SVV BAC using this protocol has been previously described [29]. Briefly, we used a recombinant plasmid encoding red fluorescent protein (RFP) interrupted by the kanamycin gene (kindly provided by Dr. Benedikt Kaufer, Freie Universität Berlin, Germany). Using oligonucleotide primers specific for regions flanking SVV ORF63/70 at the 5’-end and RFP-specific sequences at the 3’-end, we amplified a 1748 bp DNA fragment containing RFP/kanamycin (ORF63 mRFP Fw: TACCATCTGAATGTTACGTACATAAATAAAACGCTTCTCAATGGCCTCCTCCGAGGACG, ORF63 mRFP Rev: GACAGGGGTAACATGTTAGCGGCTCCCTATTGGGTAAGGGACTACAAGGCGCCGGTGGAG). The DNA fragment was used to transform E. coli GS1783 containing wild-type SVV BAC. We selected kanamycin-resistant colonies and extracted recombinant BAC DNA and confirmed recombination using Hind III digestion and agarose gel electrophoresis. We identified the recombinant BAC clones that contained RFP/kanamycin in place of SVV ORF63 and eliminated the kanamycin cassette. Complete replacement of SVV ORF 63 sequences by RFP was confirmed by sequence analysis.

DHFR domains were introduced at the C-terminus of ORF63/70 using a plasmid containing the destabilization domain dihydrofolate reductase (DHFR) derived from E. coli (kindly provided by Dr. Thomas Wandless, Stanford University, California). We introduced the kanamycin-cassette at a unique restriction site (PmeI) within the sequences encoding DHFR. We amplified DHFR/kanamycin with primers specific for SVV ORF63/70 (ORF70 DHFR Fw: CCATCTGAATGTTACGTACATAAATAAAACGCTTCTCAATGATCAGTCTGATTGCGGCGTTAGCGGT, ORF70 DHFR Rev: CCATCTGAATGTTACGTACATAAATAAAACGCTTCTCAATGATCAGTCTGATTGCGGCGTTAGCGGT) by PCR and transformed of E. coli GS1783 containing mutant SVV BAC in which SVV ORF63 was replaced with RFP. After elimination of the kanamycin cassette, mutant SVV BAC in which DHFR was fused at the amino terminus of SVV ORF70 was identified by HindIII digestion and gel electrophoresis. Proper fusion of DHFR to SVV ORF70 was confirmed by sequence analysis. The recombinant BAC was purified and used to transfect Vero cells. Infected cells were grown in the presence of 10 μM trimethoprim (TMP) to stabilize the ORF70-DHFR fusion protein. SVV plaques expressing eGFP and RFP were identified and isolated using a fluorescent microscope. Sequentially, mutant SVV was passaged four to five times in Vero-CRE cells. Passaging the virus allowed recombination of ORF70-DHFR to ORF63 location, which lead to the loss of RFP. In addition, BAC vector and eGFP sequences within the virus are flanked by loxP sites, thus passing the virus in Vero cells stably expressing cre recombinase resulted in the elimination of these non-viral sequences [39]. SVV plaques that were negative for both eGFP and RFP were purified and transferred from Vero-CRE cells to TRFs via serial passage. DNA extracted from Vero cells infected with SVV mutant was used for sequence analysis to confirm proper fusion of DHFR to the C-termini of both ORF63 and ORF70.

### Wild type and mutant SVV growth curves

*In vitro* growth curves for wild type and mutant SVV were generated as described [41]. Briefly, a monolayer of uninfected Vero cells in 25 cm^2^ tissue culture flasks were infected with approximately 5X10^2^ Vero cells previously infected with either wild type or ORF63-DHFR SVV. At 3, 24, 48, 72, 96, 120, 144 and 168 hours p.i. cells were trypsinized, diluted and seeded on triplicate dishes containing uninfected Vero cells. After approximately one week, infected cells were stained with crystal violet and infectious plaques were counted.

### Accession numbers

SVV Delta, ORF63/70-DHFR SVV, SVV ORF63 gene, and SVV ORF32 gene: GenBank NC_002686. VZV pOka and VZV ORF63 gene: GenBank AB097933.

## Results

### SVV inhibits type I IFN-induced ISG expression

To determine if SVV interferes with type I IFN-mediated responses, we studied IFN-stimulated response element (ISRE)-dependent transcription in SVV-infected luciferase reporter cells. We used telomerized rhesus fibroblasts (TRFs) stably expressing firefly luciferase under the control of the ISRE as well as constitutively expressing renilla luciferase to control for differences in cell viability between the samples. TRF-ISRE cells were infected with SVV.eGFP at a ratio of 5:1 (uninfected to SVV-infected cells) and, after 42 hours, incubated with rhesus or human IFNα for 6 hours. Productive virus infection was confirmed by visualizing eGFP expression using immunofluorescence microscopy. The firefly and renilla signal was measured and the ratio of these values reflected ISRE activity. In mock-infected cells, incubation with increasing concentrations of rhesus IFNα corresponded with increased ISRE activity. However, only a minimal response to IFNα was observed in SVV-infected cells (Fig 1A, left panel). The rhesus reporter cells were also activated by human IFNα and a comparable reduction in ISRE activity was observed in the SVV-infected cells (Fig 1A, right panel). A dose-dependent increase of luciferase activity in SVV-infected cells was not due to the presence of uninfected cells since this was also observed when the infected cells were sorted for high GFP expression by flow cytometry before IFN-treatment (Fig 1B), suggesting that high concentrations of IFN can partially overcome the inhibition by SVV. The ISRE element drives the expression of interferon stimulated genes (ISG). To study if SVV inhibits IFNα-induced ISG-expression, we infected TRFs with SVV.eGFP for 40 hours and incubated with increasing concentrations of recombinant universal type I IFN (uIFN) for 8 hours. Productive SVV infection was confirmed by the detection of SVV ORF63 expression (Fig 1C). Expression of ISG15, ISG54 and Mx-1 was observed in all mock-infected IFNα-stimulated samples, but was absent in SVV-infected cells (Fig 1C). These data show that SVV inhibits IFNα-mediated activation of ISRE-dependent reporter gene expression and ISG protein expression.

### SVV blocks IFN-mediated nuclear translocation of STAT by preventing STAT2 phosphorylation

**Fig 2:**
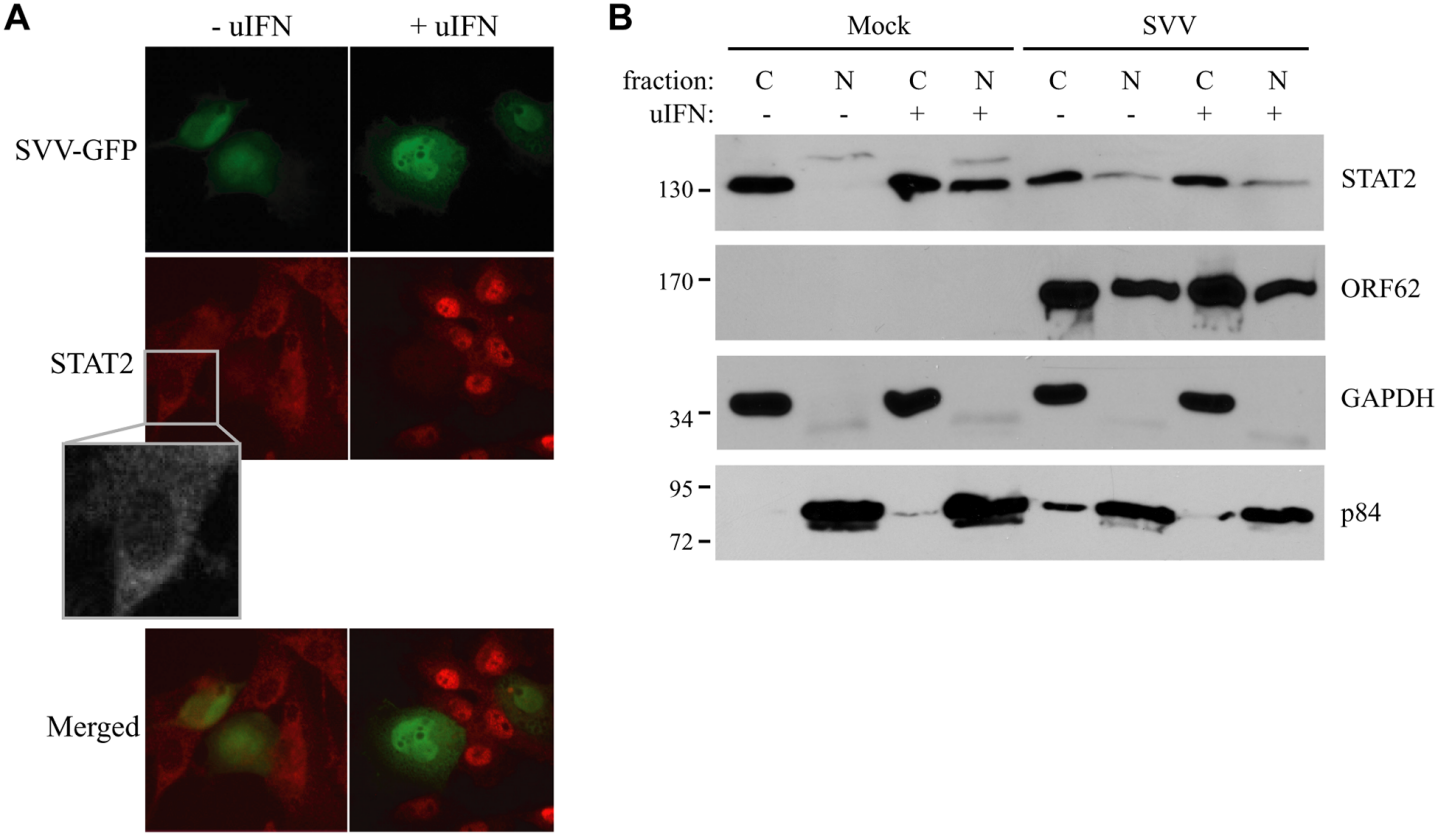
IFN-induced nuclear translocation of STAT is blocked in SVV-infected cells. (A) TRFs were infected with SVV.eGFP (ratio 10:1) and at 48 hours p.i., the cells were stimulated with 5000 U/ml uIFN for 40 minutes. Cells were fixed with 4% paraformaldehyde, permeablized and stained for STAT2 using a specific antibody. SVV infection (green) and STAT2 localization (red) were visualized by immunofluorescence microscopy. Insert shows enlargement of the outlined area. (B) Cytoplasmic and nuclear fractions were isolated from mock- and SVV.eGFP (ratio 5:1)-infected Vero cells that were stimulated with 5000 U/ml uIFN for 40 minutes at 48 hours p.i.. The fractions were analyzed for STAT2 expression by SDS-PAGE and western blotting. An antibody directed against ORF62 confirmed productive SVV-infection. Fraction purity was confirmed using GAPDH (cytosolic) and p84 (nuclear). Results from one of three independent experiments is shown.

The engagement of IFN with the IFN-receptor results in the activation of the JAK-STAT signal transduction pathway. The resulting phosphorylation of STAT1 and STAT2 allows their heterodimerization and association with IRF9, forming the ISGF3 complex that subsequently shuttles to the nucleus to initiate ISRE-dependent transcription [42]. The nuclear translocation of the ISGF3 complex is thus essential for ISRE activation. We analyzed IFN-induced nuclear localization of STAT in SVV-infected cells. TRFs were infected at a 10:1 ratio with SVV.eGFP and incubated with uIFN for 40 minutes at 48 hours post infection (p.i.). In uninfected cells, STAT2 was found predominantly in the cytosol in the absence of IFN and in the nucleus upon IFN-treatment (Fig 2A). In contrast, STAT2 was not translocated to the nucleus in SVV-infected cells (green/eGFP) upon IFN-treatment (Fig 2A). In addition, we isolated cytoplasmic and nuclear fractions of SVV.eGFP-infected Vero cells (ratio 5:1) and determined the cellular localization of STAT2 by western blot. Separation of cytosol and nuclei was confirmed using GAPDH and the nuclear matrix protein p84 (Fig 2B). In uninfected cells, STAT2 was found in the cytosolic fraction in the absence of IFN-treatment, whereas stimulation with uIFN led to the redistribution of STAT2 to both cytoplasmic and nuclear fractions. In contrast, STAT2 remained predominantly cytosolic in SVV-infected cells even upon IFN-treatment (Fig 2B). The SVV ORF62 protein was found in both cytoplasmic and nuclear fractions. This distribution is consistent with reports for the homologous VZV protein that, while primarily nuclear during early times of infection, localizes to the cytoplasm at later times of infections as a results of phosphorylation by ORF66 [43, 44]. Thus, in an asynchronous infection one would expect both nuclear and cytoplasmic expression of ORF62. Taken together, these data suggest that SVV inhibits IFN-dependent ISG-induction by abrogating the IFN-associated translocation of STAT2.

**Fig 3:**
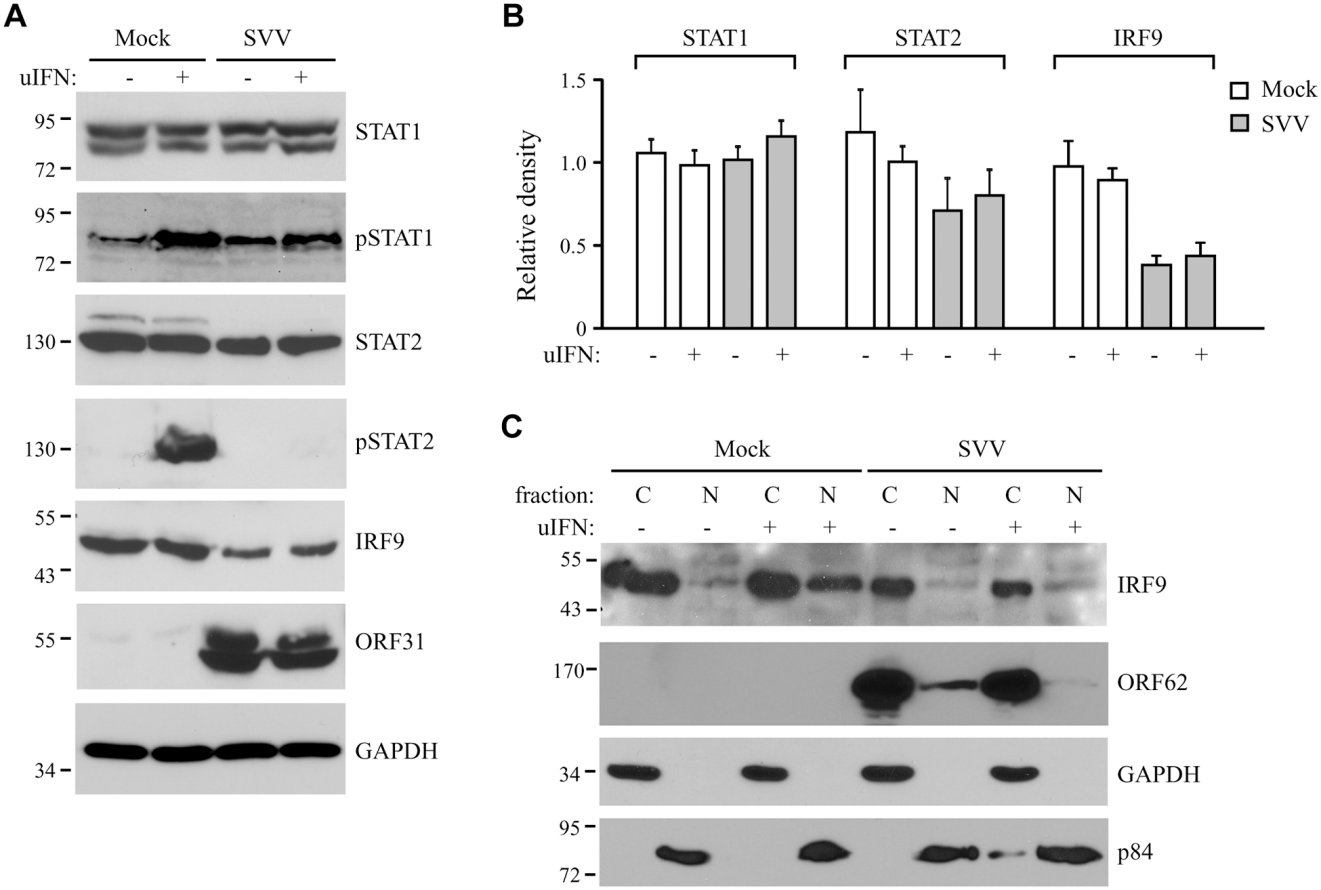
SVV prevents IFN-mediated phosphorylation of STAT2 and reduces expression levels of STAT2 and IRF9. (A) TRFs were infected with SVV.eGFP (ratio 5:1) for 48 hours and stimulated for 20 minutes with 5000 U/ml uIFN. Lysates were analyzed for expression of various members (non-phosphorylated as well as phosphorylated (p)) of the JAK-STAT pathway using SDS-PAGE and western blot with antibodies specific for the indicated proteins. ORF31 expression confirmed productive viral infection and GAPDH was used as a loading control. (B) Relative expression of STAT1, STAT2 and IRF9 in SVV-infected cells compared to unstimulated mock-infected cells (set at 100%). Shown are the averages of four independent experiments. (C) Mock- and SVV.eGFP-infected cells (ratio 5:1) were stimulated with 5000 U/ml uIFN at 48 hours p.i. for 20 minutes. Cytoplasmic and nuclear fractions were isolated and analyzed for IRF9 expression by SDS-PAGE and western blotting. An antibody directed against ORF62 confirmed productive SVV-infection. Purity of fractionation was confirmed using GAPDH (cytosolic) and p84 (nuclear). Results from one of three three independent experiments is shown.

Next we examined whether the inhibition of STAT2 nuclear translocation correlated with a SVV-mediated reduction in steady state levels of ISGF3 members or impaired STAT1/STAT2 phosphorylation. TRFs infected with SVV.eGFP for 48 hours were stimulated with IFN for 20 minutes. Steady state levels and IFN-induced phosphorylation of STAT1 were comparable between SVV- and mock-infected cells (Fig 3A). In contrast, IFN-induced STAT2 phosphorylation was absent in SVV-infected cells and steady state levels of the protein also appeared to be reduced (Fig 3A). Densitometric analysis of STAT2 protein using four independent experiments confirmed an approximately 25% decrease in STAT2 levels (Fig 3B). However, this decrease was not statistically significant. In contrast, we observed a significant decrease of more than 50% in IRF9 levels by SVV (Fig 3A and 3B). Interestingly, the reductions in STAT2 and IRF9 protein levels were observed regardless of IFN stimulation (Fig 3B). Since IRF9 drives the nuclear translocation and retention of phosphorylated STAT1 and STAT2 [42], we studied the localization of residual IRF9 in IFN-stimulated SVV-infected Vero cells by analyzing isolated cytoplasmic and nuclear fractions in western blots. In control cells, IFN-stimulation triggered the increased translocation of IRF9 from the cytosol to the nucleus. However, this increased nuclear translocation of IRF9 was not observed in SVV-infected cells (Fig 3C). Furthermore, this experiment confirmed the SVV-mediated reduction in IRF9 expression levels. These data suggest that SVV abrogates JAK-STAT signaling by both preventing the phosphorylation of STAT2 and reducing STAT2 and IRF9 protein levels.

### SVV ORF63 inhibits IFN-mediated JAK-STAT signaling

**Fig 4:**
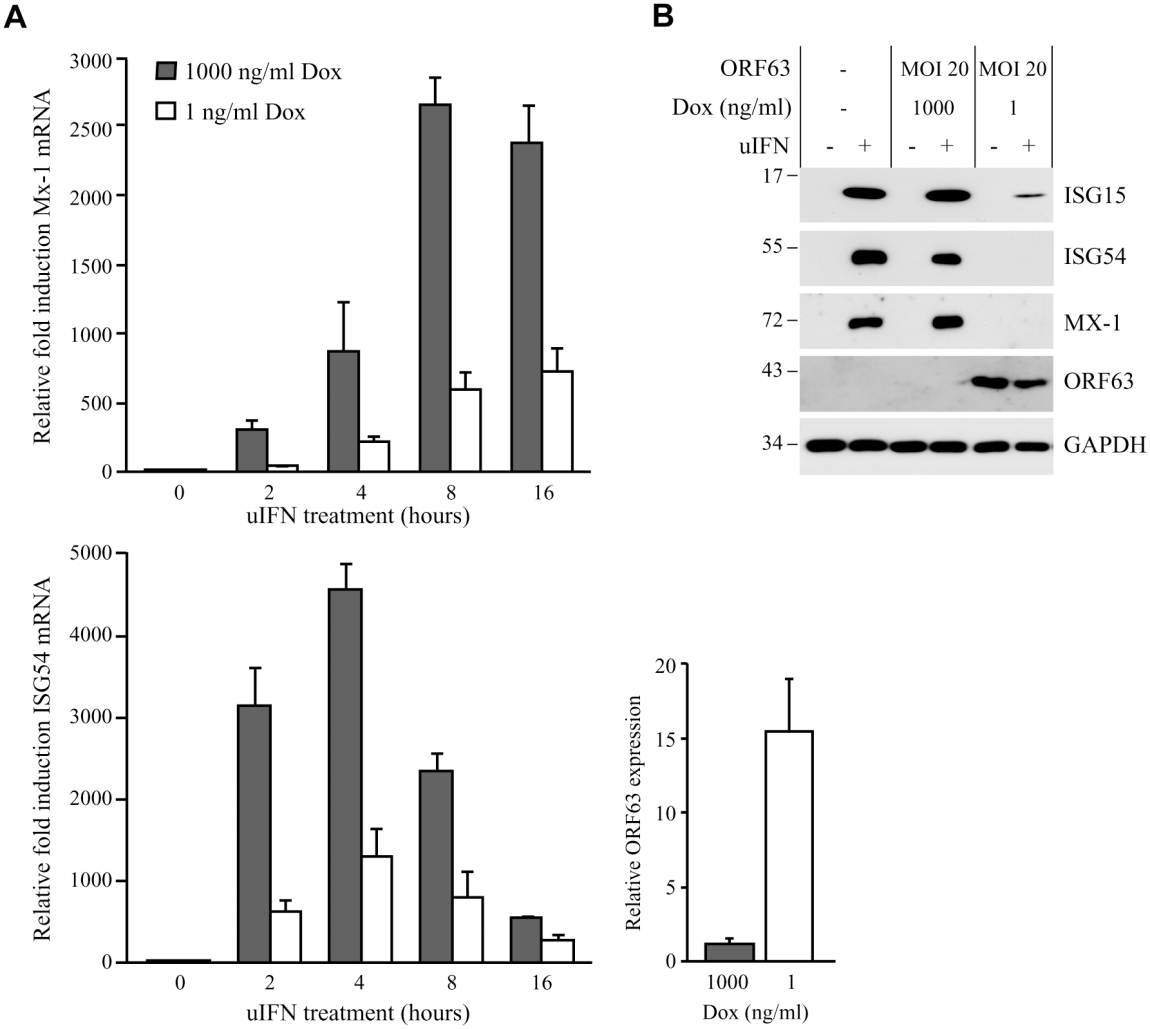
SVV ORF63 inhibits IFN-stimulated gene expression. (A) TRFs were co-infected with AdTA at MOI 10 and AdORF63 at MOI 20 in the presence of 1000 ng/ml doxycycline (Dox) to suppress ORF63 expression or 1 ng/ml Dox to allow for ORF63 expression. At 26, 34, 38 and 40 hours p.i. the cells were stimulated with 5000 U/ml uIFN for 16, 8, 4 and 2 hours, respectively, after which RNA was harvested to quantify Mx-1 and ISG54 mRNA expression by qPCR. ORF63 mRNA expression was confirmed by qPCR using ORF63-specific primers. Data were normalized to the level of GAPDH mRNA expression in each sample and are shown as fold induction relative to unstimulated control cells. Shown are the means ± standard error of the mean of two independent experiments with three replicates/sample in each experiment. (B) TRFs were mock-infected or co-transduced with AdORF63 (MOI 20) and AdTA (MOI 10) and cultured in the presence of 1000 ng/ml or 1 ng/ml Dox. 40 hours p.i. the cells were incubated with 5000 U/ml uIFN for 8 hours and lysed in SDS sample buffer. Lysates were analyzed for ISG15, ISG54 and Mx-1 expression by SDS-PAGE and western blot. ORF63 staining confirmed expression of the protein in cells treated with 1 ng/ml Dox. GAPDH was used as a loading control. One representative experiment out of three independent experiments is shown.

Ambagala *et al*. showed that wild type VZV can replicate in cells preincubated with IFNα, but an ORF63 deletion mutant could not [17]. This observation suggested that VZV ORF63 might be involved in the ability of VZV to evade IFN responses. VZV and SVV ORF63 share 52% overall amino acid homology [4, 45]. To determine if SVV ORF63 plays a role in the inhibition of IFN-stimulated responses observed in SVV-infected cells, we constructed a recombinant adenovirus expressing SVV ORF63 under the control of a tetracycline-responsive promotor (AdORF63). ORF63 expression is induced by co-infection with a recombinant adenovirus expressing the tetracycline-regulated transactivator (AdTA) [37]. These adenoviruses lack the E1-region [36], and therefore unable to interfere with IFN-signaling [46]. TRFs were co-transduced with a multiplicity of infection (MOI) of 10 of AdTA and with increasing MOIs of AdORF63. The expression of ORF63 was monitored at 48 hours p.i. (S1A Fig). Increasing MOI correlated with increasing ORF63 expression levels as expected. However, higher ORF63 levels also resulted in decreased GAPDH expression, suggesting that high expression levels of ORF63 may be cytotoxic. The transactivator expressed by AdTA can be inactivated by the tetracycline derivative doxycycline (Dox). To fine-tune ORF63 expression levels, we transduced TRFs with AdORF63 (MOI 20) and AdTA (MOI 10) in the presence of decreasing amounts of Dox. We detected robust ORF63 expression in cells that were incubated with 1 ng/ml Dox (S1B Fig) and these expression levels were comparable to ORF63 expression in cells infected with SVV (S1C Fig). At 1 ng Dox, GAPDH levels were not affected whereas lower Dox concentrations resulted in decreased GAPDH levels reflecting reduced cell viability (S1B Fig). To examine whether ORF63 inhibits IFN signaling we treated TRFs with IFN for up to 16 hours in the presence of 1ng Dox and studied ISG expression using qPCR (Fig 4A). In addition we used 1000 ng/ml Dox to inhibit ORF63 expression and under these conditions we observed increased expression of Mx-1 and ISG54 mRNA, reaching peak expression at 8 and 4 hours of IFN stimulation, respectively. In ORF63 expressing cells, however, Mx-1 and ISG54 mRNA levels were severely reduced at all time points (Fig 4A). ORF63 expression was confirmed by qPCR (Fig 4A, lower right panel). We also examined ISG protein expression by western bloting in the absence or presence of ORF63: after 8 hours of stimulation with IFN, high levels of ISG15, ISG54 and Mx-1 were detected in mock-transduced cells and in AdORF63-transduced cells treated with 1000 ng of Dox, whereas expression was absent or barely detectable in AdORF63-transduced cells treated with 1 ng Dox (Fig 4B). Taken together, these data indicate that SVV ORF63 inhibits type I IFN-induced gene expression.

### SVV ORF63 induces IRF9 degradation in a proteasome-dependent manner

**Fig 5:**
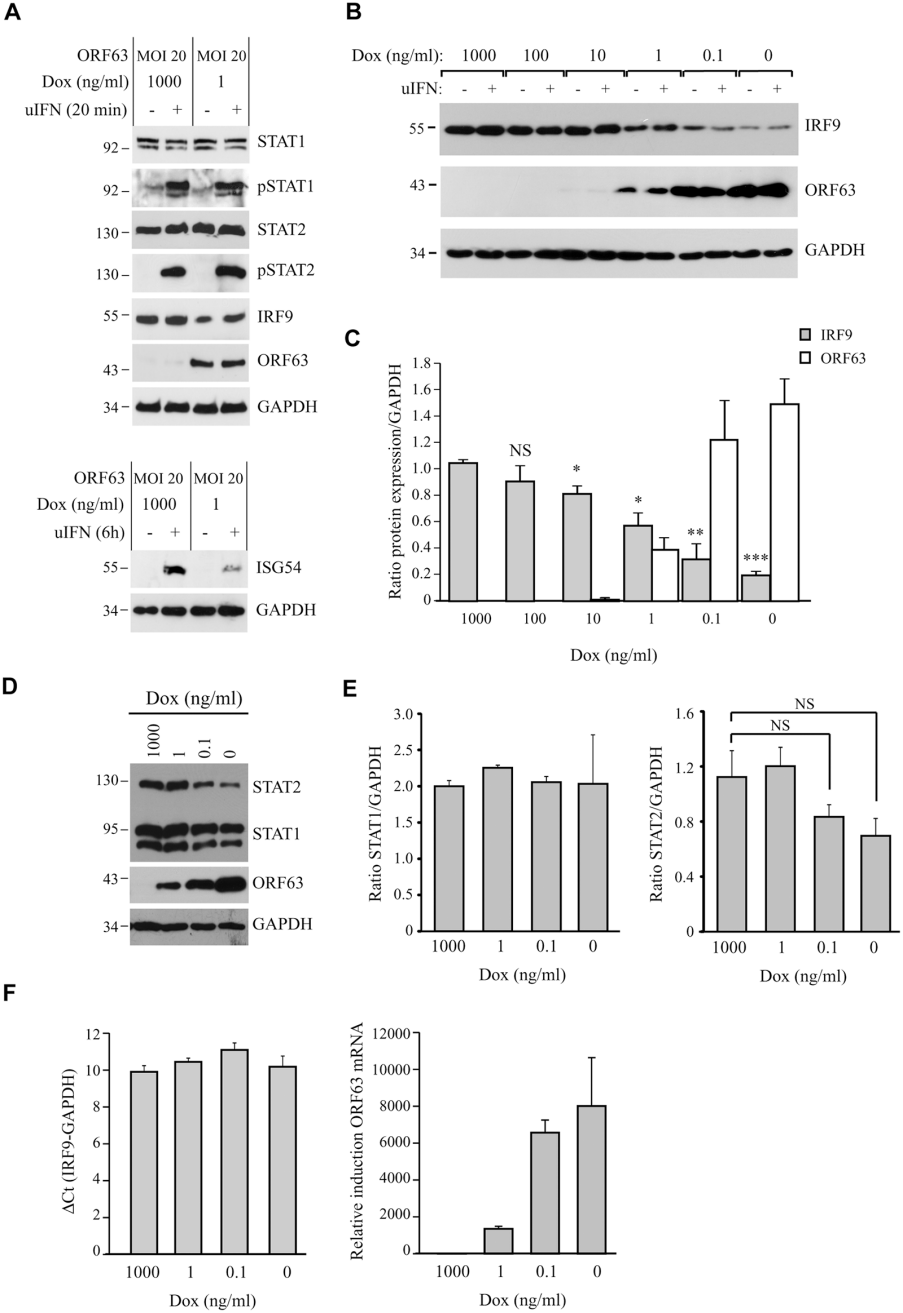
ORF63 does not affect STAT2 activation but promotes IRF9-degradation. (A) TRFs were infected with AdORF63 at MOI 20 and AdTA at MOI 10 in the presence of 1000 ng/ml Dox (control) or 1 ng/ml Dox for 48 hours. The cells were stimulated with 5000 U/ml uIFN for 20 min and lysates were analyzed via SDS PAGE and western blot for steady expression and phosphorylation status (p) of the indicated members of the JAK-STAT pathway. GAPDH was used as a loading control. ORF63 expression was confirmed using a specific antibody. One representative experiment out of three independent experiments is shown. The GAPDH signal was used a loading control. (B) TRFs were infected with AdORF63 at MOI 20 and AdTA at MOI 10 and simultaneously incubated with decreasing amounts of Dox. 48 hours p.i. the cells were stimulated with 5000 U/ml uIFN for 20 minutes, lysed and stained for IRF9, ORF63 and GAPDH. (C) Graph showing IRF9 and ORF63 expression normalized to GAPDH. Shown are the means ± standard error of two independent experiments. *: p-value < 0.005, **: p-value < 0.0005, ***: p-value < 0.00005; compared to expression in cells incubated with 1000 ng/ml Dox. (D) TRFs were infected with AdORF63 at MOI 20 and AdTA at MOI 10 and incubated with decreasing amounts of Dox. 48 hours p.i. the cells were lysed and stained for STAT1, STAT2, ORF63 and GAPDH. (E) Graphs showing STAT1 and STAT2 expression relative to GAPDH expression in the same samples. Shown are the means ± standard error of two independent experiments. NS: non-significant. (F) TRFs were infected like described in D and at 48 hours p.i. RNA was harvested to quantify IRF9 and ORF63 mRNA expression by qPCR. Data were normalized to the level of GAPDH mRNA expression in each sample. IRF9 expression is shown as delta cycle threshold (ΔCt). ORF63 expression is shown as relative expression compared to cells incubated with 1000 ng/ml Dox. Shown is a representative experiment of two independent experiments with three replicates/sample each.

The inhibition of IFN-induced ISG expression by ORF63 correlated with our observations in SVV-infected cells. Since reduced STAT2 phosphorylation as well as decreased amounts of STAT2 and IRF9 proteins were observed in SVV-infected cells (Fig 3A and 3B), we examined whether expression of ORF63 leads to the inhibition of IFN-induced STAT2 phosphorylation and reduced steady state levels of STAT2 and IRF9. Expression and phosphorylation status of members of the JAK-STAT pathway were examined in AdORF63/AdTA-transduced TRFs stimulated with uIFN for 20 minutes or 8 hours. Despite an inhibition of IFN-induced ISG expression in ORF63-expressing cells (Fig 5A, lower panel) the expression levels and phosphorylation status of STAT1 and STAT2 were unchanged (Fig 5A, upper panel). However, we did observe a reduction in steady state levels of IRF9 when ORF63 was present (Fig 5A). To confirm that ORF63 affects IRF9 expression, we transduced TRFs with AdORF63/AdTA with decreasing concentrations of Dox to obtain increasing ORF63 expression levels. Western blot analyses revealed that increasing levels of ORF63 inversely correlated with decreasing IRF9 levels (Fig 5B and 5C). Interestingly, reduced IRF9 expression was observed in both unstimulated and IFN-stimulated samples (Fig 5B), suggesting that ORF63 reduces IRF9 regardless of IFN signaling. To confirm that ORF63 does not affect STAT2 levels we transduced TRFs with AdORF63/AdTA in the presence of decreasing amounts of Dox. We observed a reduction in STAT2 levels in cells expressing high levels of ORF63 (0.1 and 0 ng/ul Dox) (Fig 5D). However, STAT1 and GAPDH expression were also affected in these samples, but when we normalized STAT1 and STAT2 expression to GAPDH expression the reduction in STAT1 or STAT2 levels was not significant when ORF63 was expressed (Fig 5E). To determine whether ORF63 affects the transcription of IRF9 we studied IRF9 mRNA levels in TRFs transduced with AdORF63/AdTA in the presence of decreasing amounts of Dox. However, we did not observe a reduction of IRF9 mRNA levels upon increasing transcription of ORF63 suggesting that ORF63 does not affect IRF9 transcription (Fig 5F).

**Fig 6:**
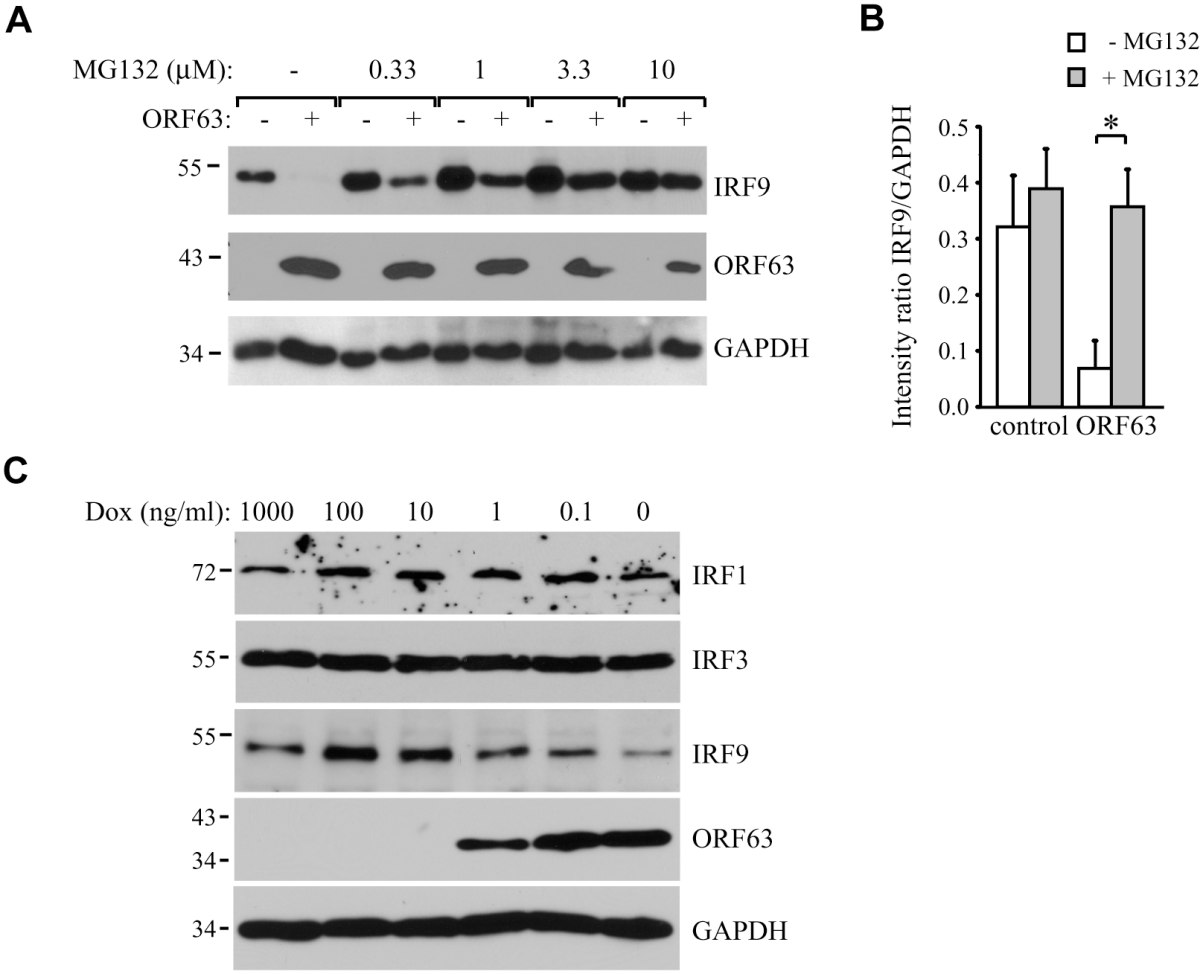
ORF63 induces proteasomal-degradation of IRF9, but does not affect IRF1 or IRF3. (A) TRFs were infected with AdORF63 at MOI 20 and AdTA at MOI 10 in the presence of 1000 ng/ml Dox (-) or 1 ng/ml Dox (+) for 48 hours. During the last 16 hours of infection cells were incubated with increasing concentrations the proteosomal inhibitor MG132. Lysates were stained for IRF9 and ORF63 levels by SDS-PAGE ans western blot. The GAPDH signal was used a loading control. (B) The ratio of IRF9 to GADH expression in MG132-treated (10 μM) control and ORF63-expressing cells is presented. Shown are the means ± standard error of the average of 4 independent experiments, * indicate P-value of <0.01. (C) TRFs were infected with AdORF63 at MOI 20 and AdTA at MOI 10 in the presence of the indicated amounts of Dox. At 48 hours p.i. cells were lysed and stained for the indicated proteins using specific antibodies. The GAPDH signal was used a loading control.

To analyze if ORF63 promotes IRF9 degradation via the proteasome we incubated ORF63-expressing cells with increasing concentrations of the proteasome inhibitor MG132 for 16 hours prior to lysing the cells. Treating ORF63-expressing cells (+) with increasing concentrations of MG132 reversed IRF9 degradation, but we also observed a slight increase in IRF9 levels in control cells (-) (Fig 6A). To determine if the rescue of IRF9 expression in ORF63-expressing cells was due to reduced turnover of residual IRF9 or due to actively blocking ORF63-mediated IRF9 degradation, we averaged the ratio of IRF9 and GAPDH expression in MG132-treated (10 μM) control or ORF63-expressing cells in four independent experiments (Fig 6B). While MG132-treatment increased IRF9 expression in control cells, the difference was not statistically significant. In contrast, MG132-treatment of ORF63-expressing cells resulted in a fivefold increase in IRF9 expression (Fig 5B, p = 0.007). From these data we conclude that ORF63 promotes IRF9 degradation in a proteasome-dependent manner. In addition to IRF9, related IRF proteins play an important role in the regulation of the expression of IFN and ISGs [47–49]. To determine whether SVV ORF63 affects the expression of IRFs other than IRF9, we monitored the steady state expression of IRF1 and IRF3 in TRFs transduced with AdORF63/AdTA in the presence of decreasing amounts of Dox. Increasing levels of ORF63 reduced IRF9 expression levels, but not that of IRF1 or IRF3 (Fig 6C). While we cannot formally rule out that SVV ORF63 might affect other IRFs it seems likely that SVV ORF63 specifically induces the degradation of IRF9 and thus preventing IFN-mediated ISG induction.

### IRF9 degradation by SVV ORF63 is responsible for inhibition of ISG expression

**Fig 7:**
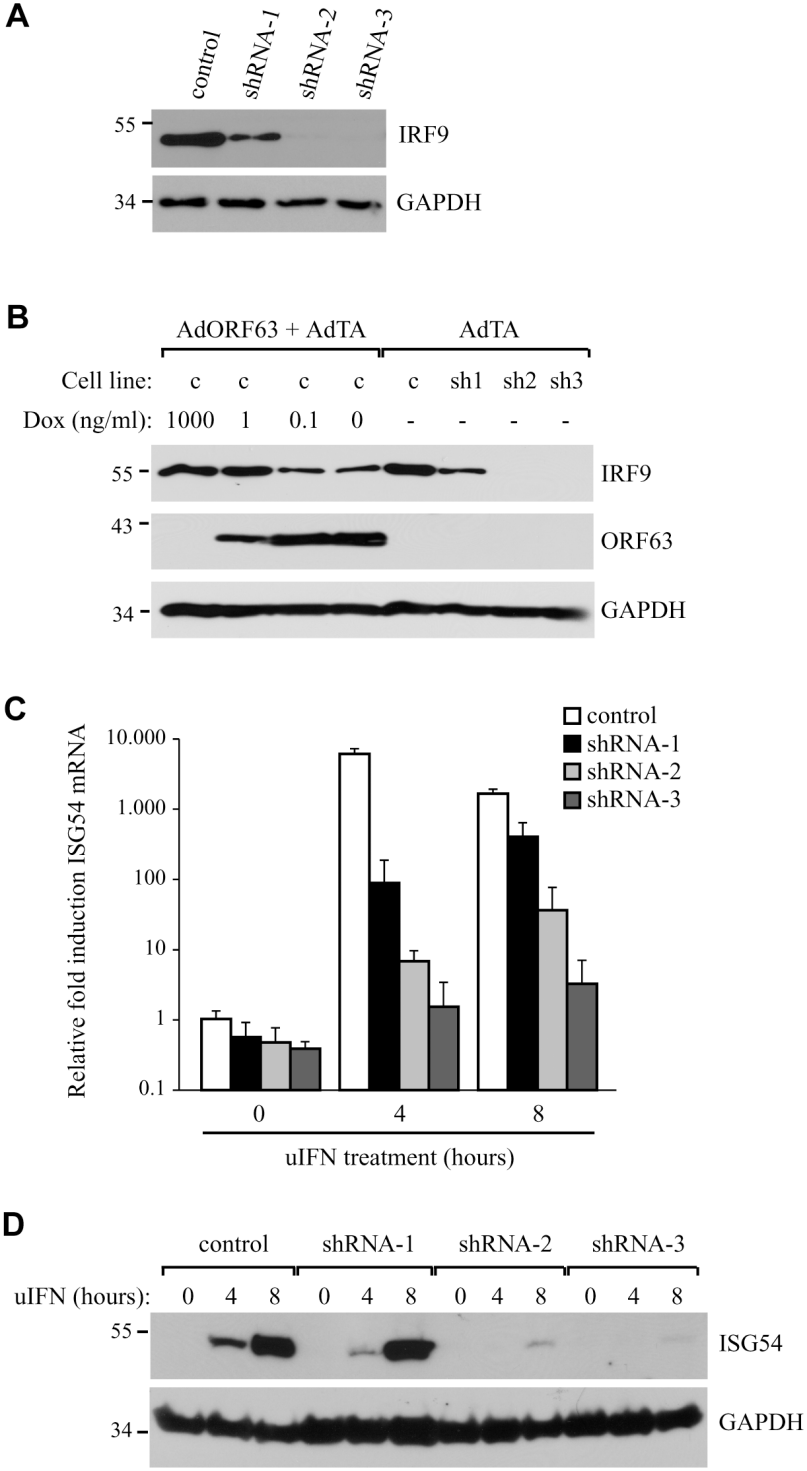
IRF9 is required for efficient ISG induction and overexpression of IRF9 overcomes JAK-STAT inhbition by ORF63. (A) IRF9 expression levels were analyzed in THF-ISRE control cells and cells expressing shRNA V3LHS 322329 (shRNA1), V3LHS 322332 (shRNA2) or V2LHS 69847 (shRNA3) by SDS PAGE and western blot. (B) THF-ISRE cells were infected with AdORF63 at MOI 20 and AdTA at MOI 10 in the presence of the indicated amounts of Dox. THF-ISRE control and shRNA-expressing cells were infected with AdTA at MOI 30. At 48 hours p.i. all cells were lysed and ORF63 and IRF9 levels in the lysates were determined by SDS-PAGE and western blot using specific antibodies. GAPDH was used as a loading control. (C) RNA was isolated from THF-ISRE expressing IRF9-specific siRNAs that were stimulated for 4 or 8 hours. RT-PCR and qPCR were used to determine ISG54 expression in all cells. Data were normalized by the level of GAPDH mRNA expression in each sample and are shown as the relative fold induction. Shown are the means ± standard error of three replicates. One of two representative experiments is shown. (D) THF-ISRE cells expressing the IRF9-specific siRNAs were stimulated with 1000 U/ml uIFN for 4 or 8 hours and lysates were analyzed for ISG54 expression using SDS-PAGE and western blot.

To determine whether the reduction of IRF9 was sufficient to prevent ISG-induction, we attempted to recapitulate the ORF63-effect by reducing IRF9 levels using small hairpin RNA (shRNA). Telomerized human fibroblasts (THF) stably expressing ISRE-luciferase (THF-ISRE) were transduced with lentivectors expressing IRF9-specific shRNA. Western blots of IRF9 showed that IRF9 expression levels were reduced in cells expressing shRNA-1 and completely absent in cells expressing shRNA-2 and -3 (Fig 7A). To compare steady state levels of IRF9 upon translational inhibition by shRNA to that of post-translational degradation by ORF63, we transduced THF-ISRE cells with AdORF63/AdTA in the presence of decreasing amounts of Dox. In these human cells, optimal ORF63 levels were observed at 0.1 ng/ml Dox or in the absence of Dox without reduction of GAPDH levels whereas only partial induction of ORF63 was observed at 1 ng Dox. A reduction of IRF9 levels consistent with ORF63 expression was observed. Reduced steady state levels of IRF9 were comparable to the partial reduction of IRF9 observed in shRNA-1 expressing cells while IRF9 was completely absent from shRNA-2 and 3 expressing cells (Fig 7B). However, when the THF-ISRE cells expressing the three shRNAs were incubated with uIFN for 4 or 8 hours, ISG54 mRNA expression was only partially reduced in the presence of shRNA-1, whereas shRNA-2 and shRNA-3 largely prevented ISG induction (Fig 7C). Analysis of IFN-induced ISG54-expression by western blotting confirmed these results (Fig 7D). These data demonstrate that removal of IRF9 is sufficient to inhibit ISG-expression and they are consistent with ORF63 affecting IRF9 protein turnover rather than IRF9 transcription or translation.

**Fig 8:**
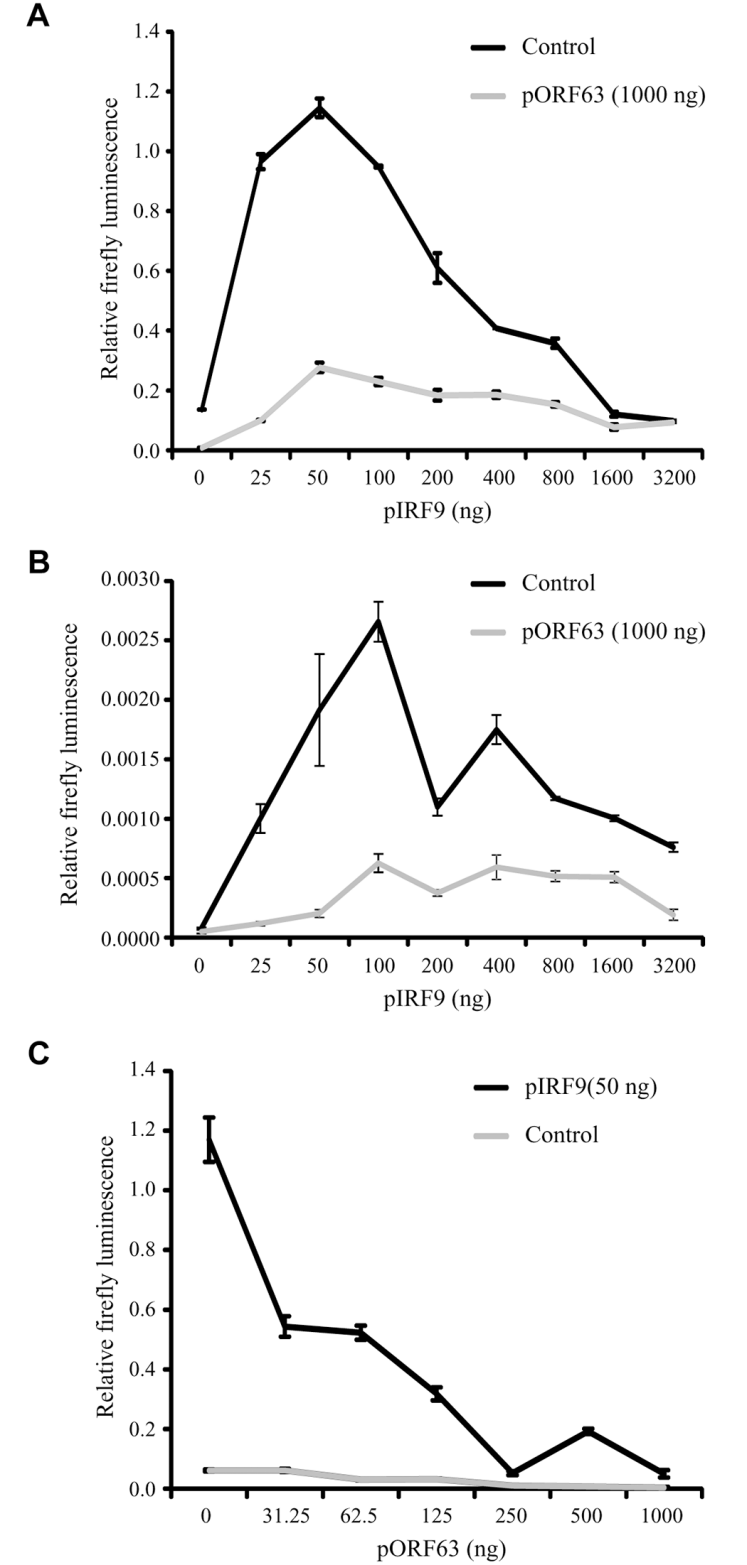
ORF63 inhibits IRF9-enhanced JAK-STAT signaling in HEK 293T cells. HEK 293T cells were co-transfected with 1 μg of a plasmid encoding ISRE-luciferase, and the indicated amounts of plasmids encoding SVV ORF63 (pORF63) and rhesus IRF9 (pIRF9). At 24 hours post transfection, luciferase expression was measured. In A and C the cells were stimulated with 5000 U/ml uIFN for 6 hours before measuring luciferase activity. The relative firefly luminescence was determined by calculating the level of firefly luciferase per cell. One representative experiment of two or three independent experiments is shown.

To further determine whether IRF9 is the primary JAK/STAT-associated target of ORF63, we took advantage of the fact that HEK 293T cells do not respond efficiently to type I IFN unless IRF9 is overexpressed [50]. Co-transfection of a plasmid encoding an ISRE-luciferase reporter with increasing amounts of rhesus IRF9-expressing plasmid resulted in an IRF9-dependent increase of luciferase expression upon treatment with IFN at 24 hours post transfection (Fig 8A; black lined graph). Interestingly, optimal luciferase stimulation was observed with 25–100 ng of the IRF9 plasmid whereas higher IRF9 concentrations resulted in decreased luciferase activity. This decrease might be due to the fact that higher IRF9 levels activate ISRE activity even in the absence of IFN-treatment (Fig 8B), and a prolonged stimulation might induce negative regulators of IFN signaling [51]. Co-transfection of 1 μg ORF63-expressing plasmid resulted in the inhibition of IRF9-induced luciferase expression both in the presence or absence of IFN (Fig 8A and 8B; gray lined graphs) suggesting that ORF63 inhibition cannot be overcome by increasing IRF9 levels. However, ORF63 needs to be in excess of IRF9 for complete inhibition since complete inhibition of IRF9-dependent ISG-induction was only observed when at least 250 ng of ORF63 plasmid was co-transfected with 50ng IRF9-expressing plasmid (Fig 8C). Together with the finding that ORF63 inhibited IRF9-dependent ISRE-transcription even in the absence of IFN-stimulation (Fig 8B) these data further support the conclusion that degradation of IRF9 is the major mechanism by which ORF63 inhibits JAK/STAT signaling.

### Inhibition of JAK-STAT signaling by a conditionally ORF63/ORF70-expressing mutant virus

**Fig 9:**
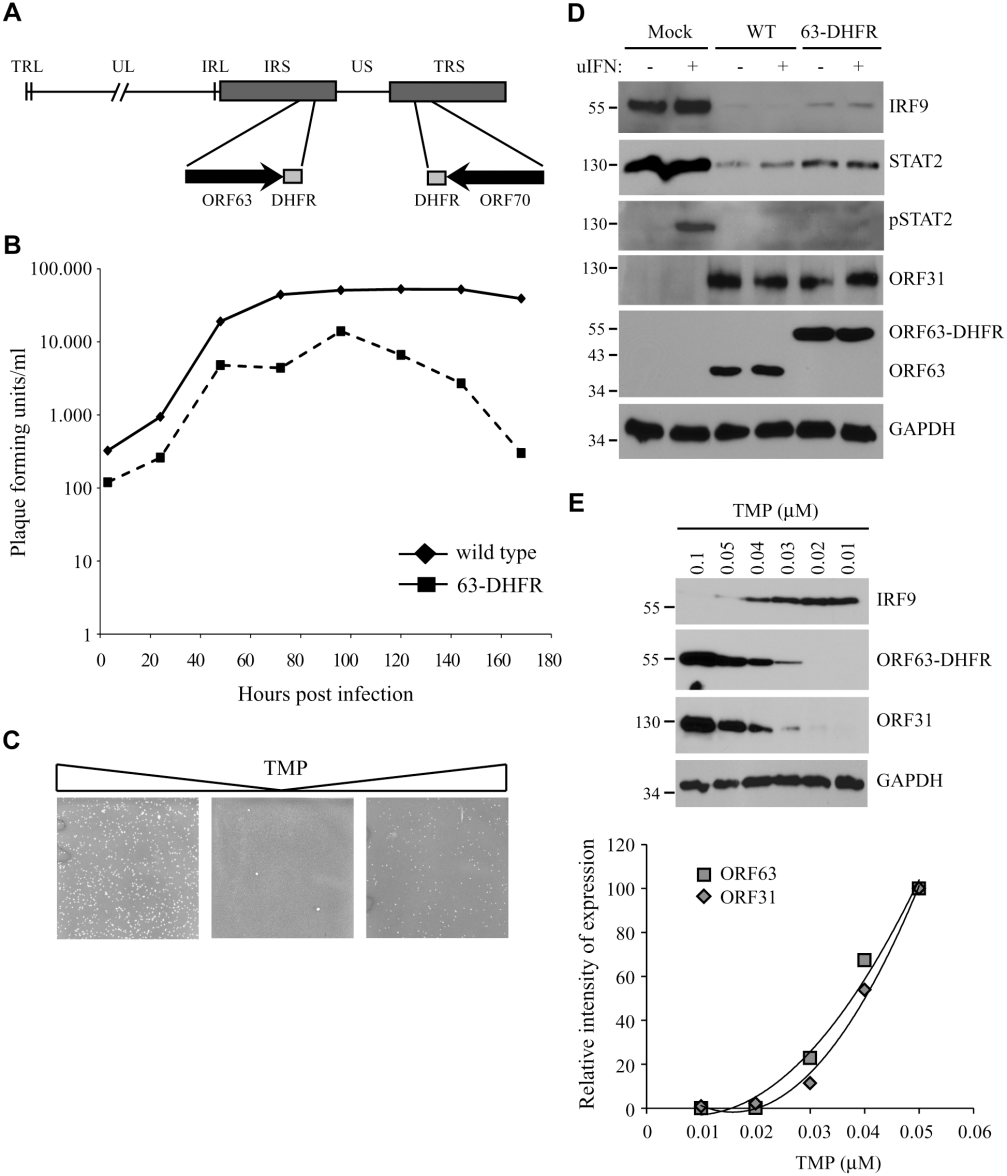
The characteristics of a conditionally ORF63/ORF70-expressing mutant virus. (A) The SVV genome consists of unique long (UL) and a unique short (US) segments and each of them being bound by inverted repeat sequences, TRL, IRL and IRS and TRS respectively. The left end of the SVV genome contains an additional invert repeat sequence. Using a recombinant SVV BAC we generated an SVV mutant in which we fused the destabilizing dihydrofolate reductase (DHFR) to the C-terminus of both ORF63 and ORF70 (63-DHFR). (B) Mono layers of Vero cells were infected with wild type or ORF63/70-DHFR SVV in the presence of 10 μM TMP and harvested at 3, 24, 48, 72, 96, 120, 144 and 168 hours p.i. The titer of each virus was determined at each time point by plaque assay. (C) Vero cells infected with ORF63/70-DHFR SVV were cultured in the presence of 10 μM TMP (first panel), after which the virus was passaged two times on Vero cells in the absence of TMP. In the third panel, TMP was added again to the culture. Viral plaques were identified by staining with crystal violet. (D) TRFs were infected with wild type and ORF63/70-DHFR SVV for 48 hours and stimulated with 5000 U/ml uIFN for 20 minutes. Cells were lysed and expression of IRF9, STAT2 and phosphorylated STAT2 (pSTAT2) was confirmed by SDS-PAGE and western blot using specific antibodies. Lysates were specifically stained for ORF31 and ORF63 to confirm infection. GAPDH was used a loading control. (E) TRFs were infected with ORF63/70-DHFR SVV for 48 hours in the presence of the indicated concentrations of TMP. Expression of IRF9, ORF63 and ORF31 were detected SDS-PAGE and western blot using specific antibodies. GAPDH was used as a loading control. One of three independent experiments is shown.

To study the role of ORF63 in the context of virus-induced inhibition of JAK-STAT signaling we constructed a conditionally ORF63/ORF70-expressing mutant using a recombinant bacterial artificial chromosome containing the complete SVV genome (SVV BAC) [39]. Recently, using the SVV BAC an ORF63/ORF70 SVV mutant was constructed by introducing stop codons in both genes. These mutations severely affected replication of the virus *in vitro* consistent with ORF63 being essential for viral growth [29]. To generate a mutant virus in which expression levels of ORF63 could be conditionally regulated, we fused the destabilizing domain (DD) of dihydrofolate reductase (DHFR) to the C-termini of ORF63 and ORF70 using two-step red-mediated mutagenesis (Fig 9A) [40]. The addition of DD-DHFR to any protein results in rapid proteasomal degradation of the fusion protein unless DHFR is stabilized with trimethoprim (TMP) [52]. We were able to recover DD-DHFR-tagged SVV in the presence of TMP and growth curves confirmed that viral growth was reduced by about 50% during the first 96 hours of infection compared to that of unmodified BAC-derived SVV when grown in 10 μM TMP (Fig 9B). A more severe reduction in viral growth was observed beyond that time point. To study the effect of TMP-removal on ORF63/70-DHFR SVV replication, we infected Vero cells in the presence of 10 μM TMP until viral plaques were detected (Fig 9C, left panel). Removal of TMP and passaging ORF63/70-DHFR SVV-infected Vero cells resulted in very few plaques (Fig 9C, middle panel). However, virus in these cultures could be rescued when 10 μM of TMP was added back to the culture (Fig 9C, right panel). These observations confirmed that the virus expressing DD-DHFR-tagged ORF63/ORF70 was able to replicate in tissue culture in the presence of TMP and viral growth can be regulated by removal of TMP. To determine whether DHFR-fusion to ORF63/ORF70 affected viral inhibition of the JAK-STAT pathway, we infected TRFs with wild type or ORF63/70-DHFR SVV in the presence of 10 μM TMP and studied expression of STAT2 and IRF9 as well as phosphorylation of STAT2 after 20 minutes of stimulation with IFN (Fig 9D). We observed a reduction in STAT2 and IRF9 expression levels as well as a complete inhibition of STAT2 phosphorylation in both wild type- and ORF63/70-DHFR-infected cells, indicating that the evasion of JAK-STAT signaling was not affected by the presence of the DHFR domains (Fig 9D). Since the removal of TMP from the culture media triggers degradation of ORF63-DHFR and ORF70-DHFR, we infected TRF with ORF63/70-DHFR SVV and cultured the cells in varying concentrations of TMP. We observed a dose-dependent decrease in ORF63 expression and ORF63 was no longer detectable when the virus was grown in 0.02 μM or less TMP (Fig 9E). This reduction in ORF63 levels led to increased levels of IRF9, indicating that expression of these proteins was inversely correlated (Fig 9E). However, the expression of ORF31 (glycoprotein B) was also affected by reducing TMP concentrations, similar to ORF63 expression (Fig 9E, western blot and graph). This result revealed that ORF63/70 is required for ORF31 expression and most likely for the expression of other SVV genes as well [24]. Restoration of IFN-sensitivity could therefore not be unequivocally assigned to the absence of ORF63 since SVV ORF31 was absent as well.

### The JAK-STAT evasion strategies employed by SVV are conserved for VZV

**Fig 10:**
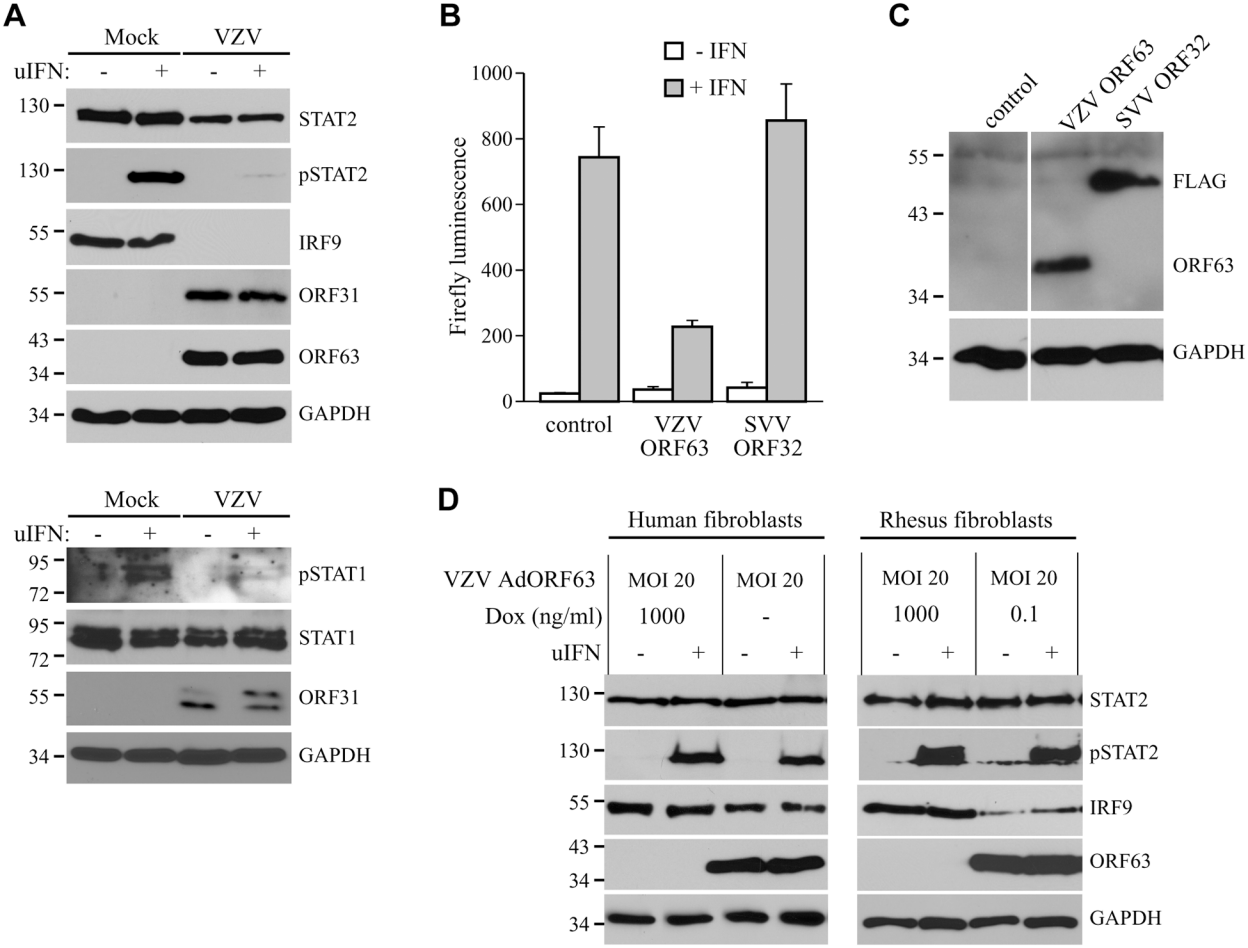
Inhibition of IFN-induced JAK-STAT signaling by VZV. (A) MRC5 cells were infected with VZV.eGFP (ratio 5:1) for 48 hours and stimulated for 20 minutes with 5000 U/ml uIFN. The lysates were analyzed for expression of STAT1, phosphorylated STAT1, STAT2, phosphorylated STAT2 (pSTAT2) and IRF9 using SDS-PAGE and western blot with specific antibodies. Lysates were stained for ORF31 and ORF63 to confirm viral infection and GAPDH was used as a loading control. (B/C) HEK 293T cells were co-transfected with plasmids encoding ISRE-luciferase and the indicated viral proteins. At 42 hours post transfection, the cells were stimulated with uIFN for 6 hours after which luciferase expression was measured. At this time part of the cells were lysed and expression of the transfected proteins was verified by SDS-PAGE and western blot using FLAG- and ORF63-specific antibodies. GAPDH was used as a loading control. (D) MRC5 cells and TRFs were co-infected with VZV AdORF63 at an MOI of 20, and AdTA at an MOI 10. The infected cells were incubated with the indicated concentrations of Dox. At 48 hpi the cells were stimulated with 5000 U/ml uIFN for 20 minutes. The lysates were analyzed for expression of STAT2, phosphorylated STAT2 (pSTAT2), IRF9, ORF63, and GAPDH by SDS-PAGE and western blot using specific antibodies.

In the experiments described above, we established that SVV inhibits JAK-STAT signaling by interfering with IFN-induced phosphorylation of STAT2 and by modulating the degradation of STAT2 and IRF9. Previous reports have shown that VZV interferes with IFN-mediated signaling [12, 17], however, the evasion mechanisms involved in this inhibition are largely unknown. To determine if VZV inhibits JAK-STAT signaling similar to SVV, we infected human fibroblasts, MRC-5 cells, with VZV.eGFP for 48 hours and activated JAK-STAT signaling by incubating the cells with uIFN for 20 minutes prior to harvesting the cells. Similar to SVV, expression levels of IRF9 and STAT2 were reduced in VZV-infected cells (Fig 10A, upper panel). Moreover, complete inhibition of IFN-induced STAT2 phosphorylation was observed, indicating that VZV employs mechanisms of JAK-STAT evasion that are comparable to those of SVV (Fig 10A, upper panel). However, unlike SVV (Fig 5A), IFN-induced STAT1 phosphorylation is blocked by VZV (Fig 10A, lower panel). To determine whether VZV ORF63 prevents ISG-induction, we co-transfected HEK 293T cells with the ISRE-luciferase reporter plasmid and plasmids encoding VZV ORF63 or, as a control, FLAG-tagged SVV ORF32. The cells were stimulated with uIFN for 6 hours at 42 hours post transfection, after which ISRE-luciferase expression was measured. In contrast to untransfected controls or SVV ORF32, expression of VZV ORF63 caused a reduction in IFN-induced luciferase expression (Fig 10B). Expression of the viral proteins was confirmed by western blot (Fig 10C). To assess if VZV ORF63 plays a role in the reduction of IRF9 expression observed in VZV-infected cells (Fig 10A), we expressed the viral protein using the tetracycline-inducible adenovirus system described in Fig 4. MRC5 cells were co-transduced with VZV AdORF63 and AdTA, incubated with the indicated concentrations of Dox and after 48 hours steady state levels of IRF9 were analyzed using western blot. We observed a prominent decrease in IRF9 expression levels in cells that expressed VZV ORF63 (Fig 10D, left panel). To determine if the reduction in STAT2 expression levels and phosphorylation observed in VZV-infected cells was due to VZV ORF63, we stimulated the transduced MRC5 with uIFN for 20 minutes to activate the pathway and studied STAT2 by western blot. STAT2 steady state levels and phosphorylation were unaffected by the presence of VZV ORF63 (Fig 10D, left panel). The same experiment was performed in TRFs and we observed a VZV ORF63-induced reduction of IRF9, but not of STAT2, in those cells as well (Fig 10D, right panel). Taken together these data show that SVV and VZV employ similar mechanisms to interfere with JAK-STAT signaling and that the ORF63 proteins of both viruses contribute to this inhibition by mediating the degradation of IRF9. VZV ORF63 induced IRF9 degradation in both MRC5 and TRFs (Fig 10D), indicating that the pathway’s inhibitory target is conserved between human and rhesus cells.

## Discussion

The data presented here show that both SVV and VZV inhibit IFN-mediated ISG induction and reduce the expression of IRF9. In addition, we observed a marginal decrease in STAT2 levels and a complete inhibition of IFN-dependent phosphorylation of STAT2 in both SVV- and VZV-infected cells. Since IRF9 is essential for JAK-STAT signaling, the degradation of IRF9 by ORF63 is thus part of a multi-pronged inhibition of IFN-mediated antiviral gene induction. These observations are consistent with previous reports showing that VZV prevents induction of Mx-1 in brain fibroblasts and reduces STAT2 levels in infected brain fibroblasts [53]. We further demonstrate that SVV ORF63 promotes the degradation of IRF9 in a proteasome-dependent manner, but does not affect STAT2 protein levels or its phosphorylation. SVV ORF63 and VZV ORF63 share 52% amino acid identity and there is evidence that these proteins are expressed in latently infected ganglia [5, 21, 22, 28, 45, 54–56]. We observed that inhibition of IFN-signaling by both proteins correlated with degradation of IRF9 whereas neither protein affected STAT2 expression or phosphorylation. Together with the previous report by Ambagala *et al*. demonstrating IFN-sensitivity of ORF63-deficient VZV [17] our data thus strongly suggest that ORF63 plays a central role in IFN-signaling inhibition by both SVV and VZV. In addition, our observations reveal that both viruses likely encode additional ORFs responsible for the inhibition of STAT2 phosphorylation and the reduction of STAT2 expression.

The reduction in IRF9 levels observed in SVV/VZV-infected cells and ORF63-expressing cells did not result from reduced transcription (Fig 5F and S2A Fig). Rather, ORF63-mediated the proteasomal degradation of IRF9 independently of IFN-signaling. Although this is the first description of IFN-evasion by IRF9 targeting for a herpesvirus, IRF9 (p48) targeting has been demonstrated for several other viruses. The non-structural protein (NSP) 1 of the simian rotavirus strain SA11-4F was shown to induce proteasomal degradation of all IRF proteins that contain an IRF association domain (IAD), which include IRF3, IRF5, IRF7 and IRF9 [57]. In contrast, our data suggest that ORF63 targets IRF9, but not IRF3 which in VZV is degraded by ORF61 [15]. Adenovirus E1A protein blocks IFN-induced protein expression by reducing IRF9 expression levels, yet the mechanism of this immune evasion is unknown [46, 58]. Incidentally, the presence of E1A in HEK293 cells [59, 60] could be responsible for the requirement of exogenous IRF9 for IFN-dependent ISG-induction. In our co-transfection experiments ORF63 thus seemed to be able to eliminate IRF9 once the E1A-dependent IRF9-inhibition was breached. Additionally, the reovirus type 1 Lang μ2 protein causes nuclear accumulation of IRF9, independent of IFN-stimulation, which results in severely impaired JAK-STAT signaling [61]. Finally, the human papillomavirus (HPV) 16 E7 oncoprotein was shown to interact with IRF9 thereby preventing ISGF3 formation [62]. We did not observe an interaction between IRF9 and ORF63 in immunoprecipitations studies performed in SVV-infected and ORF63-expressing TRFs. Therefore, the exact mechanism by which ORF63 elicits the degradation of IRF9 still needs to be elucidated.

IRF9 is a key player in the JAK-STAT signaling pathway activated by type I IFN: unphosphorylated STAT2 is complexed with IRF9 and the pair continuously shuttles between the cytoplasm and the nucleus, which is driven by the nuclear localization signal of IRF9 and the nuclear export signal (NES) of STAT2 [63]. Upon activation of JAK1 and TYK2, phosphorylated STAT1 and STAT2 dimerize, which results in the loss of the NES of STAT2 [63]. The requirement of IRF9 for anti-viral immune responses was demonstrated by Kimura *et al*. using IRF9 knock out murine cells. Replication of herpes simplex virus type 1 and vesicular stomatitis virus (a rhabdovirus) was greatly enhanced in IFNα-treated cultures of infected IRF9^-/-^ cells, while the cytokine limited viral replication in wild type cells [64]. In addition, Maiwald *et al*. created a mathematical model based on experimental data that shows that IRF9 determines the peak time and intensity of type I IFN-induced responses [65]. Our data using IRF9-specific shRNA are consistent with these studies and demonstrate the requirement of IRF9 for efficient ISG induction in human cells. Whereas shRNA-expressing cells seemed to express lower levels of IRF9 than ORF63-expressing cells it needs to be considered that ORF63 needs protein expression to act on IRF9 whereas shRNAs prevent protein expression itself. Thus, we concluded that reduced IRF9 levels are responsible for inhibition of IFN-induced ISG expression in both shRNA and ORF63-expressing cells. We therefore propose that that ORF63 blocks the JAK-STAT pathway by reducing IRF9 levels. This conclusion is further supported by the ability of ORF63 to counteract IRF9-mediated ISG-induction in HEK 293T cells both in the presence and absence of IFN (Fig 8).

Since IRF9-degradation was also observed in VZV-infected and VZV ORF63-transduced cells (Fig 10) it seems highly likely that the restoration of IRF9-levels was responsible for the previously described hyper-sensitivity of ORF63-deficient VZV [17]. The VZV deletion mutant used in this study contained a truncation of ORF63 in which only the first 24 amino acids of ORF63 was expressed. This mutant did not replicate in the presence of IFNα, but was able to replicate, albeit at reduced levels, in some cell types such as the osteosarcoma cell line U2OS [17]. To determine whether IRF9-depletion would restore the ability of this VZV ORF63 deletion virus to replicate in fibroblasts we infected THF-ISRE and THF-ISRE shRNA-3 cells with the VZV-deletion mutant (kindly provided by Jeff Cohen). However, we did no observe an increased growth of this virus upon IRF9-depletion. This indicates that additional functions of ORF63, either directly affecting host pathways or indirectly via other viral proteins, contribute to the reduced growth of the deletion virus.

Introducing stop codons in ORF63 and ORF70 severely impaired the growth of SVV in IFN-deficient Vero cells [29]. Similarly, when we fused a DHFR domain to the C-terminus of both ORF63 and ORF70 to create an inducible knock out for both proteins, removal of TMP resulted not only in the degradation of ORF63 and ORF70 but also prevented expression of other SVV genes that depend on ORF63 function. Because of the requirement of ORF63 for viral replication we were unable to directly address the biological significance of IRF9-degradation for evasion of JAK-STAT signaling by these varicelloviruses. Cohen *et al*. found that cells infected with the VZV ORF63 deletion virus were highly susceptible to IFN treatment [17], suggesting that ORF63-induced degradation of IRF9 plays a prominent role in evasion of this pathway. However, since ORF63 is required for viral early gene expression and viral replication [23–25], deletion of protein could also affect expression of the as yet unknown inhibitor of STAT2-phorphorylation as well. Thus, ORF63 likely impacts IFN-resistance both directly, by reducing IRF9 expression, and indirectly by regulating the expression of other IFN-inhibitory genes. Our data are thus consistent with a multipronged inhibition of JAK/STAT signaling by SVV and VZV to ensure efficient evasion of this innate immune pathway. It is not uncommon for a virus to target a signaling pathway at multiple levels. For example, VZV codes for at least three independent strategies devoted to inhibiting IRF3-driven expression of IFNs, which including ORF61 [15], ORF47 [14] and IE62 [13]. In addition, it is conceivable that IRF9-inhibition by the immediate early gene ORF63 precedes STAT2 inhibition due to sequential expression of the respective inhibitory proteins during viral infection. The relative contribution of each inhibitory pathway during viral infection thus still needs to be elucidated.

We observed that both SVV and VZV reduce levels and phosphorylation of STAT2, whereas STAT1 levels were not affected. In contrast, reduced phosphorylation of STAT1 was only observed in VZV-infected cells. The diminished STAT2 levels did not result from reduced transcription (S2B Fig). Previous reports demonstrated diminished levels of STAT1 in VZV-infected human fibroblasts treated with IFNγ [66] and reduced STAT1 phosphorylation in VZV-infected skin xenografts in the SCIDhu model [12]. A recent report confirmed VZV-mediated downregulation of STAT2, but downregulation of STAT1 was inconsistent between the experiments and inhibition of STAT1 phosphorylation was not observed [53]. Since STAT1 is shared between type I and type II IFN signal transduction pathways, it represents an attractive target for viral innate immune evasion. However, observations concerning STAT1 expression and phosphorylation in VZV-infected cells are inconsistent (our data, [12, 53, 66]), possibly due to the use of type II IFN and different cell lines. Therefore, we cannot rule out that SVV targets the phosphorylation of STAT1 in cell types other than fibroblasts. Since STAT1 phosphorylation was not reduced in SVV-infected TRFs it is unlikely that SVV interferes upstream of STAT1/STAT2 since the binding of IFN to its receptor triggers the activation of the tyrosine kinases JAK1 and TYK2 which in turn phosphorylate STAT1 and STAT2 resulting in the formation of the ISGF3 complex [67, 68]. If SVV would target either JAK1 or TYK2 one would expect a reduction in STAT1 phosphorylation. For VZV however, this possibility cannot be ruled out since STAT1 phosphorylation is inhibited. Although SVV and VZV-infected cells displayed lower expression levels of STAT2 the remaining STAT2 was not phosphorylated. Therefore, SVV and VZV most likely affect STAT2 phosphorylation directly.

Primary VZV infection starts with respiratory mucosal inoculation [69]. Recently, type III IFNs (IFNλ1–3) have been implicated in playing an important role in limiting (herpes)viral replication in mucosal tissues [70–72]. These cytokines bind to the IL28Rα/IL10Rβ receptor complex [73, 74], which is predominantly expressed on epithelial cells [75]. Engagement of the receptor results in the activation of the JAK-STAT pathway and induction ISG expression [74]. The Kaposi’s sarcoma-associated herpesvirus protein vIRF2 was shown to inhibit both IFNα and IFNλ-mediated ISG expression, by reducing the levels of STAT1 and IRF9 [76, 77]. Similarly, the inhibition of JAK-STAT signaling by VZV is likely to block IFNλ-mediated signaling in epithelial cells, thereby immediately limiting the host anti-viral responses to allow further spreading.

In addition to inhibiting IFN-dependent signal transduction, VZV inhibits activation of the IFN-gene itself and IFN-independent ISG-induction. The VZV proteins IE62, ORF47, and ORF61 all target IRF3-mediated induction of IFNβ and ISG genes, such as ISG15, ISG54 and IS56 [14, 15, 22]. VZV ORF61 also blocks the TNFα-mediated activation of NFκB by inhibiting IκBα [16]. Taken together with our observations, it appears that multiple VZV and SVV ORFs are devoted to interfering at sequential steps along the induction of IFN and anti-viral ISG.

Despite efficiently counteracting IFN activation and IFN-dependent signaling, several reports have indicated the importance of type I IFNs in limiting VZV replication and spread *in vivo*. Children that were treated for leukemia and were suffering from varicella showed significantly reduced dissemination of the virus in response to the administration of intra-muscular IFNα [78]. In addition, experiments in severe combined immunodeficiency mice engrafted with VZV-infected human skin (SCIDhu mice) showed that preventing IFN signaling with antibodies specific for the interferon receptor resulted in larger cutaneous lesions compared to mice that were untreated [12]. These *in vivo* responses are most likely explained by the fact that the establishment of an anti-viral state prior to infection is more difficult to overcome than IFN-responses in infected cells. IFNα-treatment of melanoma cells prior to VZV infection led to a reduction in plaque formation [17]. Thus, IFN likely prevents viral spread during the acute phase of infection by inducing an antiviral state in target cells. However, further sensitizing VZV to IFN by therapeutically blocking viral IFN-evasion mechanisms could improve the ability of IFN to prevent spread and/or reactivation of VZV *in vivo*. This concept could be experimentally tested in non-human primates using the SVV model. Since ORF63 expression have been demonstrated in latently-infected ganglia of SVV-infected rhesus macaques [5], it is also conceivable that IFN evasion is essential for maintaining viral latency. Dissecting the role of ORF63 in limiting IFN signaling could thus lead to a better understanding of the role of IFN in viral latency and reactivation.

## Supporting Information

S1 Fig Expression of ORF63 using a tetracycline-inducible adenovirus. (A) TRFs were co-infected with AdTA at a multiplicity of infection (MOI) of 10 and the indicated MOI of AdORF63. At 48 hours p.i. the cells were lysed and analyzed for ORF63 and GAPDH expression via SDS-PAGE and western blot using specific antibodies. GAPDH was used to monitor cell viability. (B) TRFs were co-infected with AdTA MOI 10 and AdORF63 MOI 20 for 48 hours in the presence of decreasing concentrations of doxycycline (Dox). Cells were lysed and ORF63 and GAPDH expression was analyzed via SDS-PAGE and western blot. (C) TRFs were infected with the indicated ratios of SVV.eGFP-infected cells to uninfected cells for 48 hours or the cells were infected with AdORF63 MOI 20 and AdTA MOI 10 in the presence of decreasing amounts of Dox. At 48 hours post infection cells were lysed and lysates were analyzed on SDS-PAGE and western blot for expression of IRF9 and ORF63. Transferrin receptor (TfR) was used as a loading control.
(TIF)

S2 Fig Transcription of IRF9 and STAT2 is not affected in SVV- and VZV-infected cells. TRFs were infected with SVV.eGFP (ratio 3:1) and MRC5 cells were infected with VZV.eGFP (ratio 3:1). Complete infection was confirmed by visualizing eGFP using fluorescence microscopy. 48 hours p.i. RNA was harvested to quantify IRF9 (A) and STAT2 (B) mRNA expression by qPCR. Data were normalized to the level of GAPDH mRNA expression in each sample. IRF9 and STAT2 expression is shown as delta cycle threshold (ΔCt).
(TIF)
