# Supplementary figures and images for: Varicella Viruses Inhibit Interferon-Stimulated JAK-STAT Signaling through Multiple Mechanisms
Source: PLoS Pathog. 2015 May 14;11(5):e1004901. doi: 10.1371/journal.ppat.1004901 (PMC4431795; doi:10.1371/journal.ppat.1004901)

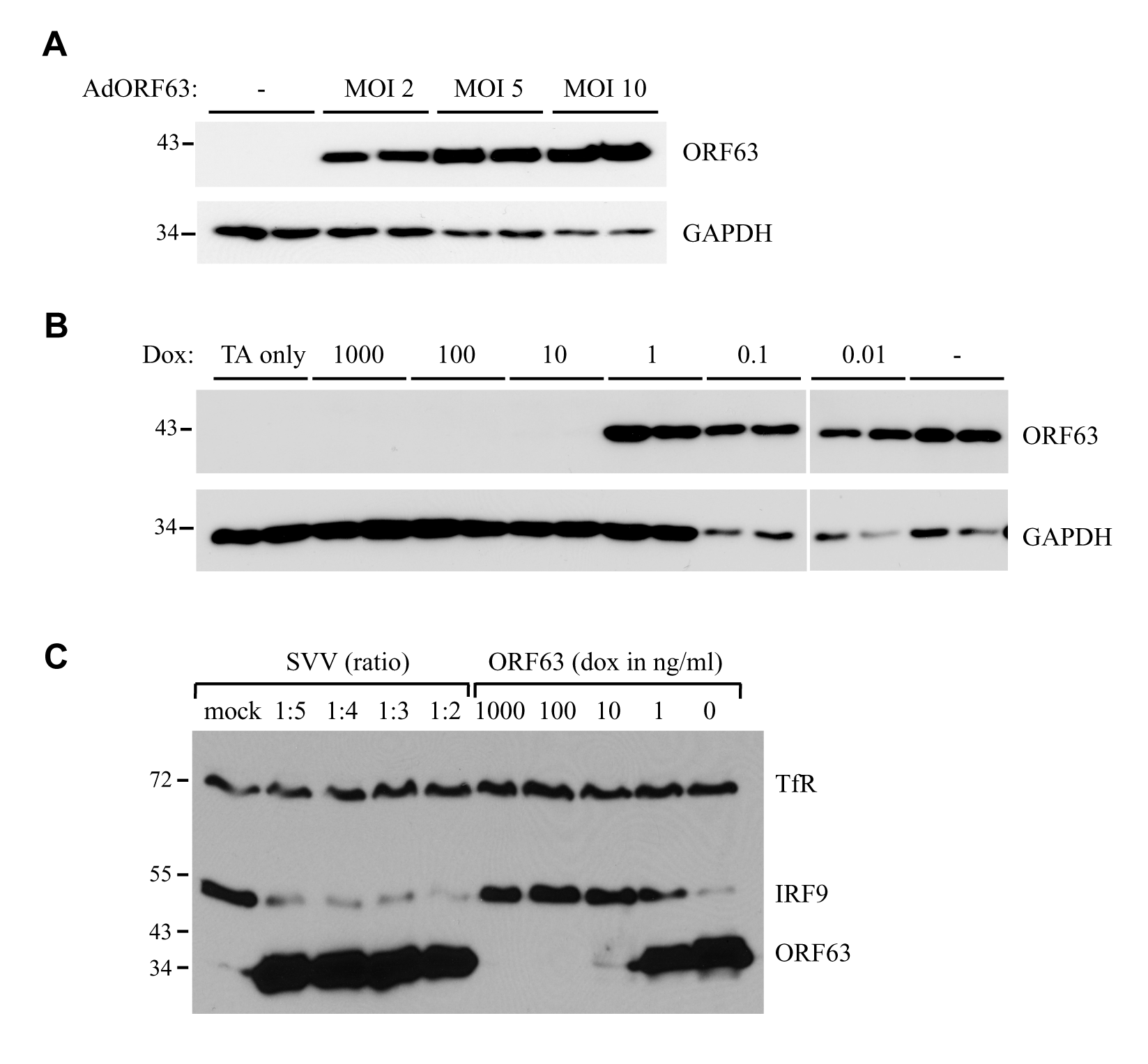

Supplement: S1 Fig — (A) TRFs were co-infected with AdTA at a multiplicity of infection (MOI) of 10 and the indicated MOI of AdORF63. At 48 hours p.i. the cells were lysed and analyzed for ORF63 and GAPDH expression via SDS-PAGE and western blot using specific antibodies. GAPDH was used to monitor cell viability. (B) TRFs were co-infected with AdTA MOI 10 and AdORF63 MOI 20 for 48 hours in the presence of decreasing concentrations of doxycycline (Dox). Cells were lysed and ORF63 and GAPDH expression was analyzed via SDS-PAGE and western blot. (C) TRFs were infected with the indicated ratios of SVV.eGFP-infected cells to uninfected cells for 48 hours or the cells were infected with AdORF63 MOI 20 and AdTA MOI 10 in the presence of decreasing amounts of Dox. At 48 hours post infection cells were lysed and lysates were analyzed on SDS-PAGE and western blot for expression of IRF9 and ORF63. Transferrin receptor (TfR) was used as a loading control. (TIF) [file ppat.1004901.s001.tif]

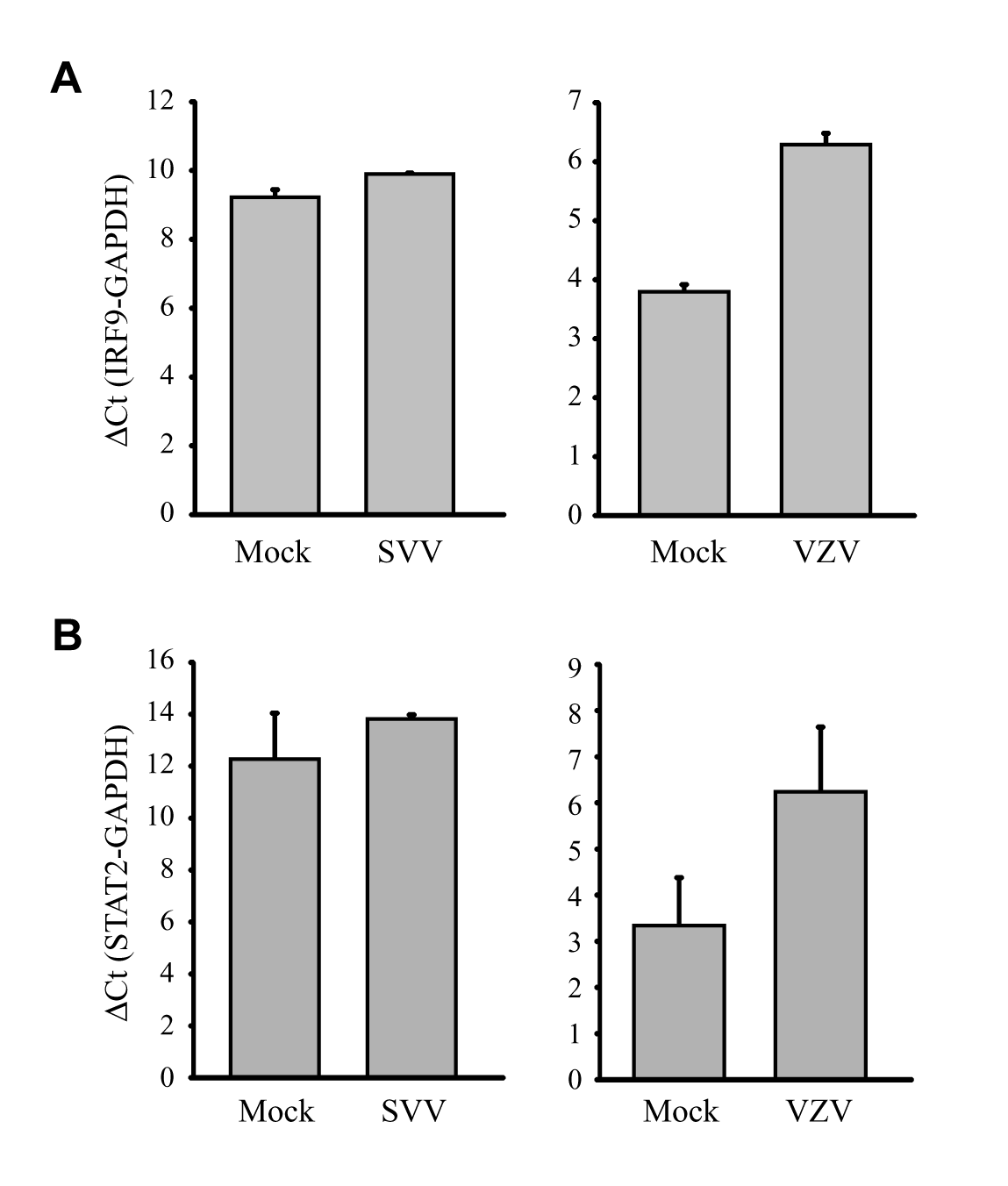

Supplement: S2 Fig — TRFs were infected with SVV.eGFP (ratio 3:1) and MRC5 cells were infected with VZV.eGFP (ratio 3:1). Complete infection was confirmed by visualizing eGFP using fluorescence microscopy. 48 hours p.i. RNA was harvested to quantify IRF9 (A) and STAT2 (B) mRNA expression by qPCR. Data were normalized to the level of GAPDH mRNA expression in each sample. IRF9 and STAT2 expression is shown as delta cycle threshold (ΔCt). (TIF) [file ppat.1004901.s002.tif]
